# Arterial Switch for Transposition of the Great Arteries: Treatment Timing, Late Outcomes, and Risk Factors

**DOI:** 10.1016/j.jacadv.2023.100407

**Published:** 2023-07-19

**Authors:** Dan-Mihai Dorobantu, Ferran Espuny Pujol, Martin Kostolny, Katherine L. Brown, Rodney C. Franklin, Sonya Crowe, Christina Pagel, Serban C. Stoica

**Affiliations:** a Children's Health and Exercise Research Centre (CHERC), University of Exeter, Exeter, United Kingdom; b Population Health Sciences, University of Bristol, Bristol, United Kingdom; c Cardiology Department, University Hospitals Bristol and Weston National Health Service Foundation Trust, Bristol, United Kingdom; d Clinical Operational Research Unit, Department of Mathematics, University College London, London, United Kingdom; e Heart and Lung Division, Great Ormond Street Hospital NIHR Biomedical Research Centre, London, United Kingdom; f Department of Paediatric Cardiology, Royal Brompton and Harefield National Health Service Foundation Trust, London, United Kingdom

**Keywords:** arterial switch, balloon atrial septostomy, hospital resource utilization, morbidity, multicenter study, reintervention, transposition of the great arteries

## Abstract

**Background:**

Reports of long-term mortality and reintervention after transposition of the great arteries with intact ventricular septum treatment, although favorable, are mostly limited to single-center studies. Even less is known about hospital resource utilization (days at hospital) and the impact of treatment choices and timing on outcomes.

**Objectives:**

The purpose of this study was to describe survival, reintervention and hospital resource utilization after arterial switch operation (ASO) in a national dataset.

**Methods:**

Follow-up and life status data for all patients undergoing ASO between 2000 and 2017 in England and Wales were collected and explored using multivariable regressions and matching.

**Results:**

A total of 1,772 patients were identified, with median ASO age of 9.5 days (IQR: 6.5-14.5 days). Mortality and cardiac reintervention at 10 years after ASO were 3.2% (95% CI: 2.5%-4.2%) and 10.7% (95% CI: 9.1%-12.2%), respectively. The median time spent in hospital during the ASO spell was 19 days (IQR: 14, 24). Over the first year after the ASO patients spent 7 days (IQR: 4-10 days) in hospital in total, decreasing to 1 outpatient day/year beyond the fifth year. In a subgroup with complete risk factor data (n = 652), ASO age, and balloon atrial septostomy (BAS) use were not associated with late mortality and reintervention, but cardiac or congenital comorbidities, low weight, and circulatory/renal support at ASO were. After matching for patient characteristics, BAS followed by ASO and ASO as first procedure, performed within the first 3 weeks of life, had comparable early and late outcomes, including hospital resource utilization.

**Conclusions:**

Mortality and hospital resource utilization are low, while reintervention remains relatively frequent. Early ASO and individualized use of BAS allows for flexibility in treatment choices and a focus on at-risk patients.

Transposition of the great arteries (TGA) is the most common cyanotic congenital heart defect, comprising 5% of all children with congenital heart disease.^1^ TGA with intact ventricular septum (TGA-IVS) has minimal mixing of the pulmonary and systemic circulations. The typical corrective procedure is an arterial switch operation (ASO), with a surgical mortality of 2% to 5%,^2^, ^3^, ^4^, ^5^ late mortality and reintervention at 20 years of 1% to 3%,^4^^,^^6^ and 14% to 20%, respectively.^4^^,^^6^ Nevertheless, data on hospital resource utilization beyond the initial ASO procedure are lacking, instead the current focus is on mortality and reintervention. Also, long-term data on TGA-IVS mostly come from single-center studies,^6^^,^^7^ while larger multicenter registries tend to be limited to early outcomes.^3^^,^^8^^,^^9^

In terms of ongoing debates on best practice, despite a consensus on early ASO and nonroutine use of balloon atrial septostomy (BAS), there is still variability in treatment choices and their reported impact on outcomes.^1^, ^2^, ^3^ Whether within the recommended age interval,^1^ timing has further impact remains unclear^10^, ^11^, ^12^, ^13^ as is the role of ASO in the first hours/days of life.^14^^,^^15^ Regarding the role of BAS, even if it is becoming less common over time with ASO at younger ages,^1^^,^^16^ BAS use is not uniform across hospitals, varying between 20% and 80% within different practices.^8^^,^^17^, ^18^, ^19^, ^20^ A potential impact of BAS on outcomes is also debated: it was reported as very protective against early mortality in one large registry,^3^ only slightly protective in another^9^ or not at all associated with early mortality in a recent report.^8^ There are further controversies on BAS use, with differing reports of neurological complications,^8^^,^^9^^,^^21^, ^22^, ^23^, ^24^ a lack of impact of BAS on discontinuation of prostaglandins,^25^ or higher hospital costs through longer stays,^8^ all overshadowed by its role in alleviating critical hypoxia, which is known to be a risk factor for pre-ASO mortality.^16^

The LAUNCHES QI (Linking AUdit and National datasets in Congenital HEart Services for Quality Improvement) project has linked multiple national databases in England, containing clinical, morbidity, and mortality data for the national cohort.^26^ Using the LAUNCHES QI dataset, we aim to address some of the current gaps in knowledge which exist in the management of TGA-IVS by: 1) describing the treatment choices and outcomes including hospital resource utilization; 2) exploring predictors for early and late mortality and reintervention; and 3) investigating the impact of BAS as first procedure followed by ASO (BAS + ASO), as opposed to ASO alone, in a subgroup with comparable characteristics.

## Methods

### National datasets and linkage

The National Congenital Heart Disease Audit (NCHDA) is the core dataset—a national audit with mandatory submission collecting procedure-based information from all congenital heart disease centers in England and Wales, which operate in a centralized manner. Data on all 96,041 patients with at least one recorded procedure between April 2000 and March 2017 were linked to: the Paediatric Intensive Care Audit Network (PICANet) for patient admissions to pediatric intensive care units (ICU); death registrations from the Office for National Statistics; and Hospital Episode Statistics routine administrative data on inpatient, outpatient, and accident and emergency (A&E) care at hospitals in England. Linkage methodology and an overview of the dataset are published elsewhere.^26^

### Patient selection and classification

**Figure 1:**
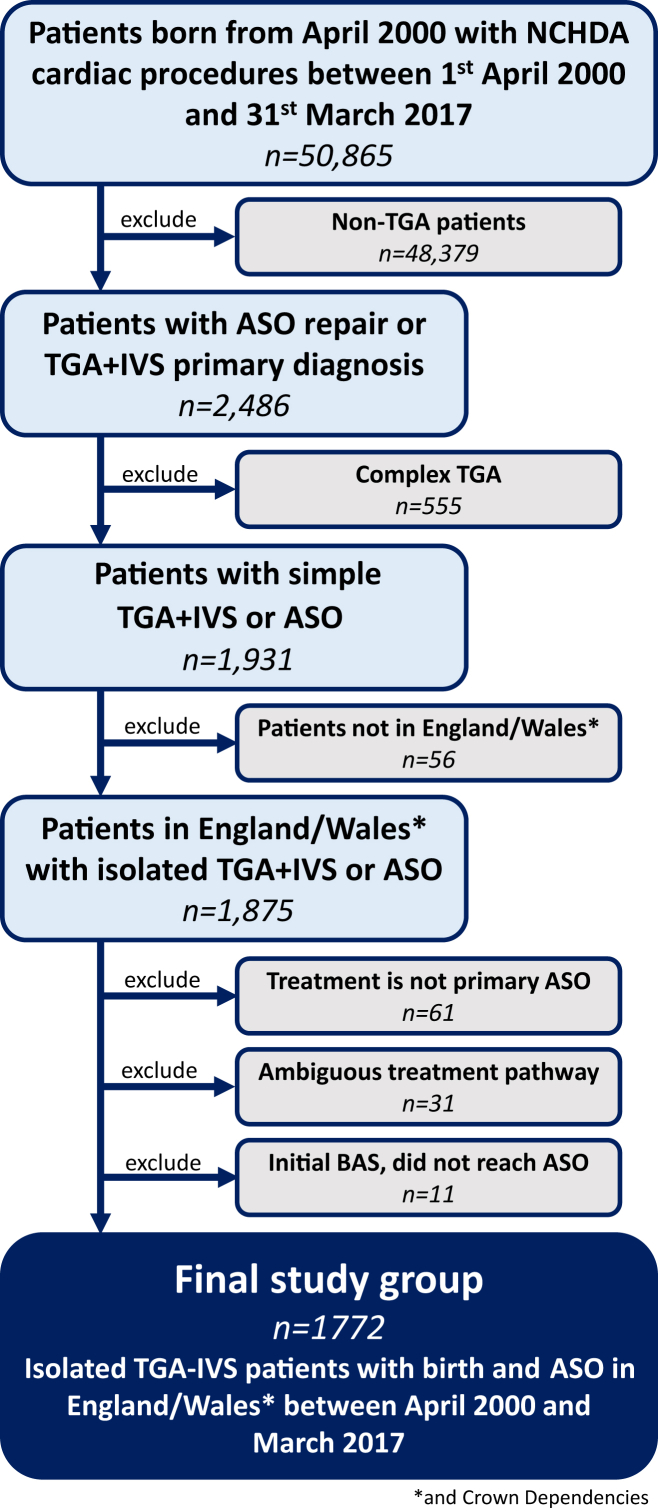
Flowchart of Study Population and Excluded Groups ASO = arterial switch operation; IVS = intact ventricular septum; NCHDA = National Congenital Heart Disease Audit; TGA = transposition of the great arteries.

The focus of this study was TGA-IVS treated by ASO (with or without an initial BAS), preceded by at most other minor cardiac procedures, with complete intervention history (born between April 2000 and March 2017) in England and Wales. Complex TGA patients and those not undergoing typical ASO repairs (such as staged or ambiguous repairs, Senning, or Mustard operations) were excluded. A minority of patients undergoing BAS with death recorded before an ASO (n = 11) were only included as part of the BAS intention to treat analysis. Deaths between BAS and ASO were excluded for the analysis of post-ASO surgery where, by definition, survival to ASO was required (and babies who died pre-ASO without a procedure would not appear in the dataset, which is procedure based). All exclusion steps are detailed in Figure 1. The corresponding diagnosis/procedure codes, specific procedure classifications,^27^ and inclusion criteria are shown in the Supplemental Methods and Supplemental Tables 1 to 3.

### Collected data and outcomes

All clinical data were organized in “care spells”, which can contain procedures, inpatient or intensive care stays, and outpatient or emergency room (A&E) visits, in any combination, at no more than 1 day apart.^26^ The following data were extracted from the LAUNCHES QI dataset.

#### Patient-related data

Sex, preterm birth, antenatal diagnosis, and congenital comorbidity.

#### Procedure-related data

Age at procedure, weight at procedure (absolute and UK age-sex weight Z score), acquired comorbidity, additional cardiac risk factors (associated cardiac conditions or complications not consisting of other congenital heart disease (CHD), such as pulmonary hypertension, cardiac dysfunction, cardiomyopathy), severity of illness marker (such as circulatory support, shock, or acidosis),^27^ cardiac procedure type, cardiopulmonary bypass duration, procedure era and center, and use of BAS.

#### Hospital resources utilization

Hospital length of stay (LoS) and ICU LoS (total, pre-ASO, and post-ASO) within the ASO procedure spell, and for the period following discharge from ASO: any and cardiac-related inpatient and ICU stays and outpatient or A&E department visits; periprocedural ICU utilization (extracorporeal membrane oxygenation [ECMO], renal support, inotrope support, and invasive/non-invasive ventilation).

#### Mortality and reintervention

In relation to the index ASO procedure (unless otherwise stated); early mortality and early cardiac reintervention (30 day and in-hospital), mid (1 and 5 years), and late (10 years) mortality and reintervention (any cardiac, surgical, transcatheter, and by anatomic subtype).

See the Supplemental Methods and Supplemental Table 4 for details on the above definitions.

### Statistical analysis

Frequencies are given as numbers (percentages), all continuous values as median (IQR), and number of records with nonmissing values are provided for each reported figure.

We performed 3 separate analyses.

1. A descriptive analysis of patient and procedure characteristics, treatment choice trends, and center variation, hospital resource utilization, reintervention, and mortality. This was done using the entire cohort, accounting for missing data (left truncation and right censoring) in each linked dataset (Supplemental Methods). The 1-, 5- and 10-year mortality and reintervention estimates were calculated using Kaplan-Meier survival analysis and conditional probability functions (taking account of competing risks) and estimates up to maximum follow-up are shown for data completeness. The median hospital resource utilization data are reported per successive year of follow-up (or month in the first year), using for each reporting period all patients having data for at least part of the period.
2. To evaluate the role of ASO timing, in relation to other patient and procedural characteristics, a multivariable regression approach was used (Cox regression for mortality and cause-specific hazards for cardiac reintervention). All models included as risk factors: age at ASO, use of BAS, and low weight (below 2.5 Kgs) at ASO. Additional risk factors that were statistically significant (*P* < 0.05) in single variable analysis (all variables listed in Table 1 were candidates) were also tested for inclusion in the multivariable models. In order to include preprocedural risk factors, this analysis was limited to those undergoing ASO in 2009 or later when such information became routinely recorded. This limited the follow-up period for this analysis to the maximum of 12 years for mortality and 7 years for reintervention.

**Table 1:** Demographic, Clinical and Procedural Data (N = 1,772)

Demographic and anthropometric	
Age at first intervention, d	3.5 (1.5-9.5)
Weight at first intervention, kg^a^	3.3 (3.0-3.7)
Age at ASO, d	9.5 (6.5-14.5)
Weight at ASO, kg^b^	3.4 (3.0-3.7)
Male	1,254 (70.8)
Preoperative clinical risk factors^c^	
Prenatal diagnosis (n = 830)	401 (48.3)
Preterm birth	36 (4.3)
Acquired comorbidity	78 (9.4)
Additional cardiac risk factors	23 (2.8)
Congenital comorbidity	26 (3.1)
Severity of illness marker	206 (24.8)
Procedure-related factors	
Septostomy before ASO	983 (55.5)
Time from septostomy to ASO, d	7.6 (5.0-11.0)
ASO bypass length, min^d^	145 (121-181)
ASO era	
2000-2004	527 (29.7)
2005-2008	412 (23.3)
2009-2012	440 (24.8)
2013-2016	393 (22.2)

Values are median (IQR) or n (%).

ASO = arterial switch operation.

a Nonmissing in n = 1,735 (774 of whom with primary ASO).

b Nonmissing in n = 1,741 (774 of whom with primary ASO).

c Reported for patients born between April 2009 and March 2017 (n = 832, except n = 830 for prenatal diagnosis).

d Reported for 1,511 patients (missing frequently before 2002, fully reported from 2010).
3. To evaluate whether the choice of primary ASO or BAS + ASO treatment strategy influences patient outcomes, a matching methodology was used. This analysis was restricted to ASO within the first 3 weeks of life, considered optimal per current guidelines.^1^ Patients undergoing BAS + ASO were matched 1:1 with patients undergoing primary ASO at the same age (±1 day), age-sex weight Z-score (±2 SDs), and during the same era (±3 years). Mortality, reintervention, and hospital resource utilization were then compared between the 2 resulting groups using a Pearson’s chi-square test for independence, Welch’s *t*-test for difference of averages, or quantile regression for difference of medians. As discussed above, patients who died between their BAS and ASO were included in this analysis.

All statistical analyses were conducted using the STATA/MP 17.0 software (StataCorp LLC) or R (R Core Team, 2014).

## Results

A total of 1,772 patients (70.8% male) with simple TGA-IVS undergoing ASO between April 2000 and March 2017 at 12 centers were included (Figure 1). There was variability in total and average case load between centers, but minimal variability by era (Supplemental Table 5).

### Descriptive analysis of demographics, treatment options, and outcomes

The overall median age at ASO was 9.5 days. Primary ASO was performed in 44.5% (n = 789) at a median age of 9.4 (IQR: 6.5-14.5) days, while 55.5% (n = 983) underwent BAS + ASO, with a median age at BAS of 1.5 (IQR: 0.5-2.5) days and median age at ASO of 10.4 (IQR: 7.4-14.5) days. Demographic, clinical, and procedure-related data are detailed in Table 1.

**Figure 2:**
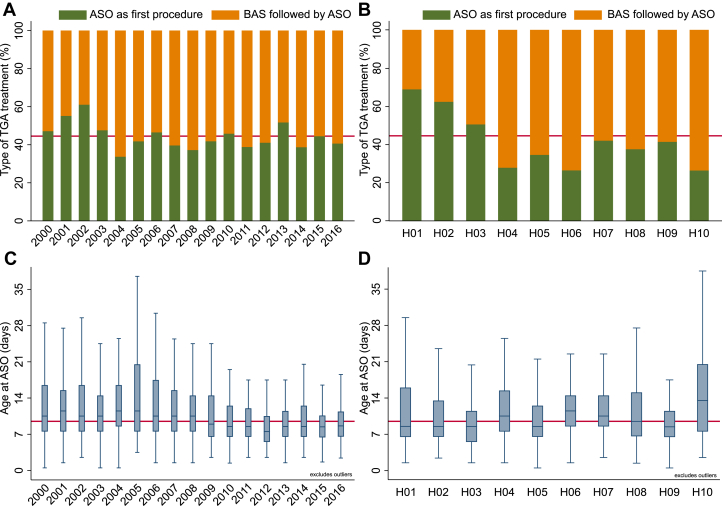
Variation in Treatment Choices and Age by Year and Center **(A)** Bar chart showing proportion of patients undergoing arterial switch operation (ASO) as first procedure and balloon atrial septostomy (BAS) followed by ASO, by treatment year. **(B)** Bar chart showing proportion ASO as first procedure and BAS followed by ASO, by treatment center. **(C)** Box and Whisker chart showing median age (IQR) at ASO by treatment year. **(D)** Box and whisker chart showing median age at ASO by center. Centers are anonymized, and 2 centers not shown due to low numbers (1 and 2 ASO Respectively). Overall average is represented by solid **red horizontal line.**

The number of ASO procedures per year ranged from 75 to 122 (median 103) with the proportion of ASO as first procedure ranging from 33.7% to 61.0% (median 41.8%), with no significant upward or downward trend after 2003 (Figure 2A). The proportion of ASO as first procedure vs BAS + ASO varied by center from 26.4% to 68.8% (Figure 2B). There was a slight decrease in median age at ASO and its range (IQR) by year (Figure 2C). Ages at ASO varied by center around the overall median of 9.5, with median values from 8.4 to 13.5 days (Figure 2D).

ASO mortality was 1.7% at 30 days (2.4% in hospital). Furthermore, 30-day and in-hospital cardiac reinterventions were 2.0% and 1.9%, respectively. Renal replacement was used in 14.0% of patients, for a median (IQR) time of 4 (2-12) days, while ECMO was used in 3.5%, with a median length of 13 (IQR: 9-17) days. For the ASO spell, median total hospital LOS was 19 (IQR: 14-24) days, ICU stay was 7 (IQR: 5-11) days, of which more time was spent after ASO compared to before. All outcomes associated with the initial ASO spell are detailed in Table 2.

**Table 2:** 30-Day and In-Hospital Survival and Reintervention, Periprocedural Resource Utilization, and Hospital Resource Utilization During the Arterial Switch Operation Hospital Spell (N = 1,772)

	Size of Reference Patient Group	Outcome Measure
30-d mortality	1,772	30 (1.7)
In-hospital mortality^a^	1,770	42 (2.4)
30-d cardiac reintervention^b^	1,738	34 (2.0)
In-hospital cardiac reintervention^c^	1,737	33 (1.9)
ICU utilization recorded in the data^d^		
ECMO used	808	28 (3.5)
ECMO support, d	28	13 (9-17)
Renal support used	809	113 (14.0)
Renal support, d	113	4 (2-12)
Inotrope support in ICU	808	802 (99.3)
Inotrope support, d	802	10 (6-18)
Invasive ventilation in ICU	1,234	1,232 (99.8)
Invasive ventilation, d	1,232	7 (4-12)
Noninvasive ventilation in ICU	935	376 (40.2)
Noninvasive ventilation, d	376	3 (1-6)
Hospital LoS		
Total (d)^a^	1,770	19 (14-24)
Pre-ASO	1,772	8 (5-11)
Post-ASO^a^	1,770	11 (8-15)
ICU LoS^d^		
Total (d)	1,422	7 (5-11)
Pre-ASO	1,422	3 (1-5)
Post-ASO	1,422	4 (3-6)

Values are n, n (%), or median (IQR).

ECMO = extracorporeal membrane oxygenation; ICU = intensive care unit; PICANet = Paediatric Intensive Care Audit Network.

a n = 2 missing discharge age.

b n = 23 died before 30 days without a reintervention; additionally n = 11 did not have at least 30 days follow-up.

c n = 32 died in hospital without reintervention, n = 2 missing age at discharge, n = 1 did not have full follow-up during stay (estimated discharge date after March 2017).

d PICANet data were available for 1,422 patients.

**Figure 3:**
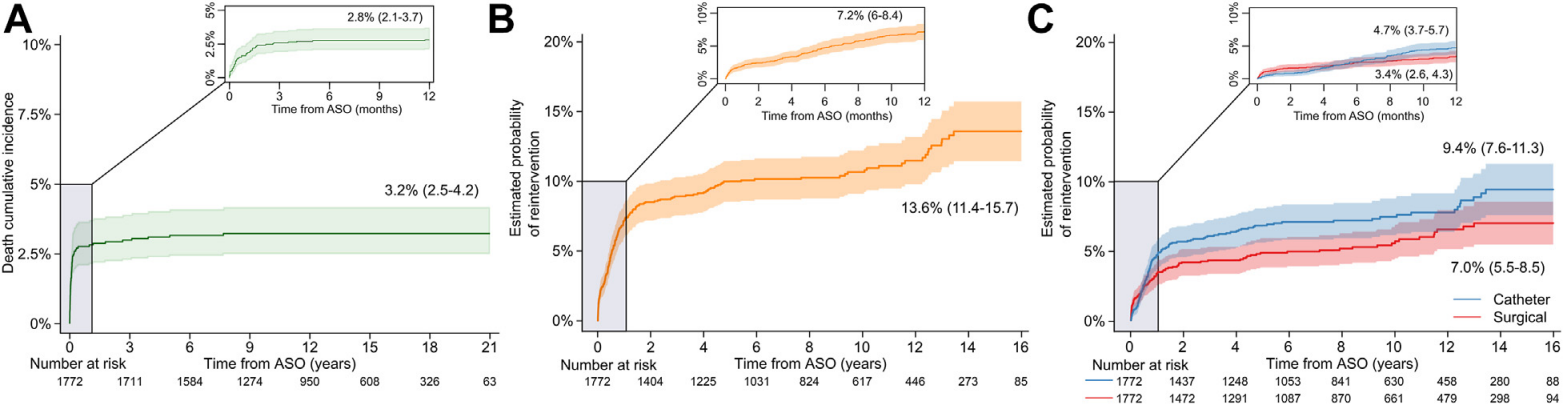
Mid and Late Outcomes after ASO in Simple TGA-IVS **(A)** Probability of death (Kaplan-Meier) over 21 years. **(B)** Probability of cardiac reintervention conditional on survival (conditional probability function) over 16 years. **(C)** Probability of cardiac reintervention conditional on survival (conditional probability function) by reintervention type (surgical or transcatheter). Survival median follow-up is 12.4 (IQR: 8.6-16.6; max: 21.8) years; reintervention follow-up is 8.2 (IQR: 4.0-12.7; max: 16.9) years. All inserts are enlarged for the first year of follow-up. All numerical data (for **A-C**) in Supplemental Tables 6 to 9. ASO = arterial switch operation; TGA-IVS = transposition of the great arteries with intact septum.

Estimated mortality at 5 years and 10 years post-ASO was 3.2% (IQR: 2.4%-4.1%) and 3.2% (IQR: 2.5%-4.2%), respectively, with most deaths occurring during the first year of follow-up (Figure 3A, Supplemental Table 6). Probability of cardiac reintervention within 5 and 10 years of ASO conditional on being alive was 10.0% (IQR: 8.5%-11.5%) and 10.7% (IQR: 9.1%-12.2%), respectively, highest incidence in the first year (Figure 3B, Supplemental Table 7). Surgical cardiac reinterventions were more frequent than catheter-based reinterventions during the first 4 months post-ASO, after which there were more catheter-based reinterventions (Figure 3C, Supplemental Tables 8 and 9).

Reintervention data were collected over a median follow-up of 8.2 (IQR: 4.0-12.7) years. During the whole follow-up period, 1 cardiac reintervention was observed in 125, 2 in 37, and 3 or more in 23. Most reinterventions were on the pulmonary arteries (either surgical or catheter based), followed by right ventricular outflow tract/pulmonary valve replacement and coronary procedures (Table 3). The probability of cardiac reintervention within 10 years of ASO, conditional on being alive, was: 9.3% (IQR: 7.5%-11.2%) for pulmonary arteries, 1.3% (IQR: 0.5%-2.0%) for right ventricular outflow tract/pulmonary valve replacement, and 1.0% (IQR: 0.4%-1.7%) for coronaries. No era effect of ASO year on cardiac reintervention risk was observed.

**Table 3:** Reinterventions After Arterial Switch Operation by Type and Sequence From Index

	First Cardiac Reintervention	Second Cardiac Reintervention	Third Cardiac Reintervention	Fourth or Later Cardiac Reintervention	Total
PA catheter^a^	88	27	17	15	147
PA surgical^b^	26	17	4	4	50
Coronary surgical^c^	13	2	0	0	15
MAPCA occlusion	9	3	1	0	13
RVOT catheter^d^	6	2	1	0	9
RVOT surgical^e^	7	1	0	0	8
Supra-aortic surgical^f^	6	1	0	0	7
Other venous procedure	4	2	0	0	6
ASD closure	6	0	0	0	6
Pacemaker	2	1	0	1	4
Atrial septostomy	2	1	0	1	4
LVOT surgical^g^	3	0	0	0	3
Other	13	3	0	0	16
Total	185	60	23	21	289

Combined procedures (n = 10) counted only once, labeled after the least frequent type (supra-aortic > LVOT > RVOT > coronary > PA). The median follow-up was 5.4 (IQR: 4.4-12.8) years.

ASD = arterial septal defect; LVOT = left ventricular outflow tract; MAPCA = major aorta-pulmonary collateral artery; PA = pulmonary artery; RVOT = right ventricular outflow tract.

a Proximal/branch balloon angioplasty ± stent implantation.

b Proximal/branch arterioplasty.

c Coronary arterial bypass procedure, reimplantation or other.

d Pulmonary valve/RVOT balloon dilation.

e Subpulmonary resection, open pulmonary valvotomy, or pulmonary valve replacement.

f Supravalvar aortic stenosis repair.

g Subaortic resection, aortic valve replacement.

**Figure 4:**
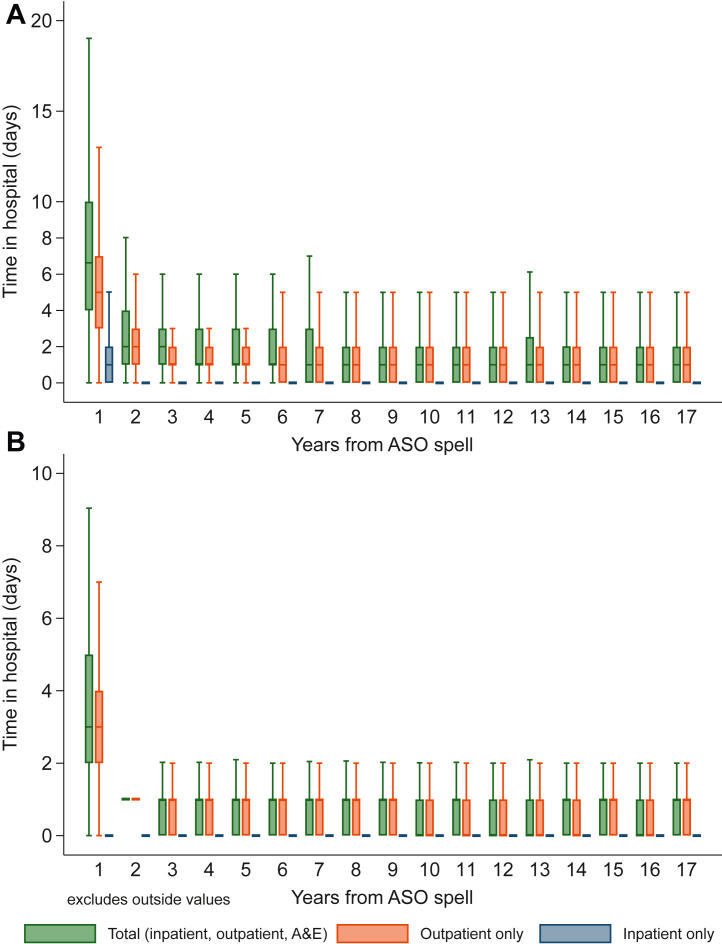
Days Spent in Hospital After the ASO Spell Ended, by Type Total (inpatient/outpatient/A&E) **(green)**, inpatient **(orange)**, or outpatient **(blue)**. **(A)** Hospital days for any reason (cardiac and noncardiac/ambiguous). numerical data in Supplemental Table 10, including split by inpatient (Supplemental Table 11) and outpatient (Supplemental Table 12). **(B)** Hospital days for cardiac reasons only. numerical data in Supplemental Table 10, including split by inpatient (Supplemental Table 11) and outpatient (Supplemental Table 12). No variations of >1 day from median were observed at year 2. The median for inpatient time in hospital was 0 (IQR: 0-0) beyond year 2. A&E = accidents and emergencies.

The median total number of days at hospital (inpatient or outpatient/A&E) was highest during the first year post-ASO discharge, at 7 (IQR: 4-10) days, and decreased gradually down to 1 (IQR: 0-2) day by 16 years after ASO, of which close to half were cardiac related (Figure 4, Supplemental Table 10). Beyond the first year of follow-up, inpatient days were uncommon (most children had no inpatient stays each year, Supplemental Table 11), with most days spent at hospital being outpatient (Supplemental Table 12). There were very few days spent in A&E without an admission (Supplemental Table 13). The median number of days spent in hospital (inpatient or outpatient/A&E) was higher in those undergoing a cardiac reintervention at any time during their follow-up (n = 183), and this was observed from the 1st to the 17th year of follow-up (Supplemental Table 14).

### Impact of ASO timing, use of BAS and low weight on mortality and reintervention

In a subgroup analysis for patients born after 2009, with complete data on comorbidities (n = 651), the age at ASO, need/use of BAS and weight at ASO <2.5 kg were not associated with early or late mortality and reintervention after ASO, in univariable and multivariable analyses (Table 4). The only factors associated with both early and late mortality in the multivariable analysis were use of ECMO (indicating the child had not recovered well from surgery) and additional cardiac risk factors (indicating more severe condition going into the switch procedure), while low weight <2.5 kg at ASO and length of invasive ventilation were only associated with higher 12-year mortality. Congenital comorbidity, longer ventilation, and ECMO at ASO were associated with higher 30-day and 7-year reintervention.

**Table 4:** Multivariable Analysis of Factors Associated With Early and Late Mortality and Reintervention After Arterial Switch Operation

	HR	95% CI	*P* Value
30-d mortality following ASO			
Age at ASO	1.03/y	1.00-1.06	0.025
BAS + ASO	3.1	0.6-15.0	0.161
Weight at ASO <2.5 kg	1.8	0.2-18.3	0.623
Additional cardiac risk factor	12.2	6.2-23.9	<0.001
Antenatal diagnosis	7.5	1.7-34.1	0.009
ECMO use at ASO	12.1	4.5-32.4	<0.001
Renal support at ASO	11.2	2.4-51.7	0.002
Mortality within 12 y from ASO			
Age at ASO	1.02/y	0.96-1.05	0.96
BAS + ASO	1.4	0.7-3.1	0.34
Weight at ASO <2.5 kg	3.8	1.5-9.5	0.005
Additional cardiac risk factor	10.0	2.8-35.7	<0.001
Antenatal diagnosis	3.5	1.5-8.6	0.005
ECMO during ASO spell	19.3	9.4-39.4	<0.001
Renal support at ASO	4.5	1.2-17.8	0.03
30-d reintervention following ASO			
Age at ASO	0.97/y	0.91-1.03	0.30
BAS + ASO	0.8	0.6-1.02	0.07
Weight at ASO <2.5 kg	0.98	0.25-3.9	0.90
Congenital comorbidity	17.9	3.2-101.4	0.001
Days of inotrope support at ASO spell	1.01/d	1.01-1.02	<0.0001
ECMO during ASO spell	21	9.3-47.8	<0.0001
Reintervention within 7 y from ASO			
Age at ASO	0.99/y	0.97-1.03	0.80
BAS + ASO	0.9	0.5-1.7	0.80
Weight at ASO <2.5 kg	0.6	0.1-3.4	0.60
Congenital comorbidity	5.2	1.6-16.8	0.006
Days of inotrope support at ASO spell	1.02/d	1.01-1.02	<0.0001
ECMO during ASO spell	3.7	1.8-7.9	<0.0001

Analysis restricted to patients undergoing ASO in 2009 or later with available covariate information (n = 651 for mortality, n = 649 for reintervention) and this is reflected in shorter follow-up than the descriptive analysis. Always in the multivariable model: age at ASO, BAS + ASO, or ASO only, weight at ASO <2.5 kg.

ASO = arterial switch operation; BAS = balloon atrial septostomy; ECMO = extracorporeal membrane oxygenation.

### Impact of BAS or ASO as first procedure in matched patient analysis

Matching BAS + ASO and primary ASO by age, weight, and procedure year resulted in a total of 174 pairs (patient characteristics comparison in Supplemental Tables 15 to 17). There were no significant differences in early, late mortality, and reintervention, or total hospital and ICU LoS, with only pre-ASO LoS being longer in the BAS + ASO group (Table 5).

**Table 5:** Comparison of Mortality, Reintervention, and Hospital Resource Utilization by Early Treatment Choice of Balloon Atrial Septostomy Followed by ASO or Primary ASO in Groups Matched by Age, Weight-for-Age (Z-Scores) and Financial Year at Procedure

	BAS Followed by ASO (n = 174)	Primary ASO (n = 174)	*P* Value
30-d mortality after first BAS/ASO, %	4 (2.3)	5 (2.9)	0.74
In-hospital mortality after first BAS/ASO, %	5 (2.9)	6 (3.4)	0.76
ASO spell hospital LoS (d)	n = 162	n = 162	
Total	22.4 (20.0-24.8)	20.0 (15.9-24.0)	0.30
Pre-ASO	9.2 (8.2-10.2)	5.3 (4.7-5.9)	<0.001
Post-ASO	13.7 (11.6-15.9)	15.1 (11.1-19.2)	0.54
ASO spell ICU LoS^a^ (d)	n = 131	n = 131	
Total	9.0 (7.4-10.6)	9.6 (6.2-13.0)	0.75
Pre-ASO	3.6 (3.1-4.2)	3.3 (2.8-3.7)	0.28
Post-ASO	5.9 (4.6-7.3)	6.9 (3.5-10.3)	0.60
Mortality (%)	n = 174	n = 174	
6 mo after BAS/ASO	4.0 (1.9-8.3)	4.0 (1.9-8.3)	1.00
21 y after BAS/ASO	4.6 (2.3-9.0)	4.6 (2.3-9.0)	1.00
Cardiac reintervention (if alive) after BAS/ASO	n = 170	n = 170	
Within 6 mo	3.5 (1.6-7.7)	4.8 (2.4-9.4)	0.39
Within 16 y	12.9 (7.3-22.2)	11.5 (6.9-18.6)	0.89
Hospital d/y after ASO spell	n = 139	n = 139	
Median (IQR) over 17 y	1 (0-3)	1 (0-3)	0.28

Values are n (%) or HR (95% CI) unless otherwise indicated. Matching restricted to those having an ASO in the first 3 weeks of life. Patients undergoing BAS that died before ASO were included in the matching pool (n = 7) and 3 were part of the matched sample. If an entry was censored, its pair was also excluded from the comparison to maintain balance.

*P* value from chi-square tests for binary outcomes (short-term mortality and significance of mortality HRs by type of repair), *t*-test with Welch formula of unequal variances for continuous outcomes (ASO spell lengths of stay), and quantile regression for median hospital d/y after ASO spell.

ASO = arterial switch operation; BAS = balloon atrial septostomy; ICU = intensive care unit; LoS = length of stay.

a Born from February 2003 onwards linked to PICANet data.

## Discussion

This detailed report of ASO in a large, national cohort of consecutive patients with TGA-IVS procedures shows low mortality and hospital resource utilization after the first year of life but relatively high reintervention rates. The patients undergoing reinterventions are also spending more days in hospital, and this was observed throughout the follow-up, regardless on reintervention timing. Markers of severe illness and low weight were associated with higher early and late mortality and reintervention, whereas age at ASO or use of BAS was not. In a matched comparison of patients treated in the first 3 weeks of life, performing a BAS as first procedure did not confer better or worse results than opting directly for an ASO, including hospital resource utilization, further clarifying the currently inconsistent results reported in the literature on this topic.^3^^,^^8^^,^^9^ These findings suggest that there is flexibility in both the age of repair and use of BAS when individualized care is offered, even if a centralized system such as in the United Kingdom does lead to a more uniform practice and higher case volumes per center. The focus of improving outcomes should be on mitigating patient risk factors and reducing the need for reinterventions, which in turn could reduce hospital resource utilization.

### Mortality, cardiac reintervention, and hospital resource utilization after ASO

Mortality and cardiac reintervention at 1 year after ASO were 2.8% and 7.2%, respectively, while at 10 years they were 3.2% and 10.7%, respectively, in line with modern international outcomes.^4^^,^^6^ This highlights that despite the relatively low mortality, reintervention is relatively high and its impact on the overall burden of care is not fully quantified, especially in terms of hospital resource use. It is still somewhat reassuring to see that most reinterventions can be achieved transcatheter (Table 3).

One CHD registry study from Utah reported <1 day on average spent as an inpatient per year, from age 2 years and beyond for TGA patients, without details on outpatient visits, or type of hospitalization.^28^ In our national cohort, beyond the first year of follow-up, hospitalizations are rare and outpatient visits appear to average one per year. Moreover, the upper quartile limit for total days spent in hospital (including outpatient visit) was at most 3 days/year and 1 day/year for cardiac causes. Even more importantly, it appears a subgroup with a high hospital stay throughout their childhood is comprised of those needing a cardiac reintervention, with the results suggesting their course is less favorable as early as the index procedure. This is important to clinicians and decision-makers, and even more so to the parents and patients, as it would suggest that for most cases, the expectation is for low burden of care throughout adolescence. On the other hand, the association between early hospital resource utilization and reinterventions is of note and would require further research to understand how it relates to other risk factors. When compared to historical series, mortality improved markedly over the past decades, but reintervention rates decreased less dramatically,^29^, ^30^, ^31^ emphasizing how those requiring reinterventions make up an ongoing critical at-risk subgroup, where further improvements are needed to improve outcomes.

### Age at ASO–impact on outcome

The median age at ASO for this group was 9.5 (IQR: 6.5-14.5) days, lower at 8.5 (IQR: 6.5-11.5) days in the most recent 5 years, in line with current recommendations^1^ and overall European practice,^2^ showing that repair in the first week of life is not always possible, even in recent years. In terms of center practice, there was little variation in median age at ASO but some differences in upper quartile threshold. This could be due to regional or institutional characteristics which we were not aiming to evaluate.

We found that the age at ASO was not associated with early or late mortality and reintervention but low weight was. This is in the context of the national UK practice to offer early ASO, which does lead to more uniform treatment age intervals. This confirms that while a complete repair as early as possible should be the aim, as recommended by current guidelines, timing can also be informed by the clinical context, such as low weight and comorbidities, and not solely by age. Antenatal diagnosis was associated to worse mortality, but this is in the context of major changes in the proportion of prenatally diagnosed TGA from <40% in 2011 to 75% in 2017 in the United Kingdom.^32^ We speculate that in the earlier years included in the multivariate analysis (starting in 2009), cases with a worse clinical course, both pre- and postnatally, might have been more likely offered prenatal screening, identified prenatally, or recoded as having prenatal screening in the patient notes. The exact underlying confounding relations are not known and as such this finding should be interpreted with caution, since the favorable role of prenatal diagnosis and rapid access to CHD care in TGA has been well established previously.^33^

### ASO or BAS first: variation in practice and impact on outcome

BAS is well established and critical in alleviating hypoxia when emergency ASO is not an option.^16^ Nevertheless, there is little guidance on when it is indicated, both in terms of timing and patient selection. This will naturally lead to differences in practice by center, as shown in our national cohort, but also in other large registries and series.^8^^,^^17^, ^18^, ^19^, ^20^ In the current study, some centers in the United Kingdom used a BAS in as much as 74% of their cases, while others in as few as 31% of theirs, without there being differences in the regional case-mix that could account for this disparate practice. This would suggest that the same case might be managed differently in different centers.

A large North American registry has investigated the impact of timing as well as use of BAS on early outcomes after ASO. It reported an almost 3-fold reduction of in-hospital mortality for those undergoing BAS, considered surprising even by the authors.^3^ Another registry of mixed TGA also showed lower mortality after BAS, but to a lesser degree (9.7% BAS vs 11.7% without BAS), and attributed this to how more complex cases already had sufficient blood mixing, and would not be chosen for BAS, increasing mortality for this group.^9^ In another recent study of more than 17,000 cases, limited to TGA-IVS, no association of BAS to early mortality was found, but longer hospital stay, higher costs, and slightly higher incidence of stroke were reported.^8^ The issue of post-BAS stroke remains unclear, with difficulties in addressing causality.^8^^,^^9^^,^^21^, ^22^, ^23^, ^24^

In our national cohort, we did not identify any impact of BAS use on early/late mortality, reintervention, and hospital resource utilization, using a multivariable analysis of consecutive patients and age/weight/era matching with ASO as first procedure. In the matched analysis, the patients undergoing BAS first had longer pre-ASO hospital stay but similar total ASO spell LoS and hospital visits over the following years. The results of our matched analysis, even if limited in scope, support primary ASO as a first choice of intervention when clinically and logistically feasible. In some reports emergency ASO helps avoid BAS in critically ill patients but with controversial benefits.^14^^,^^15^ Selective use of BAS remains an excellent option to maintain stability and temporize the surgical intervention. The exact factors driving the differences in practice are not known, and might be a combination of clinical need, logistical factors, resource availability, and team philosophy with one approach or the other.

### Study limitations

This is a retrospective analysis on linked national registries, and thus it is limited by the available data. However, the core information on CHD procedures (NCHDA) is a mandatory dataset with external audit and validation and its data quality is excellent. The limited clinical information did not allow for more detailed risk factor analysis, although comorbidities associated with CHD outcomes were evaluated.^27^ Because not all linked datasets had the same start date, and within registries not all variables were introduced at the same time, analyses had to be limited to subsets with complete data, leading to exclusions of some patients from certain analyses. Patients not undergoing at least one cardiac procedure could not be identified, so deaths without any treatment were not captured. Patient selection and classification was done through an iterative algorithm and despite review of discrepancies, some misclassification might have occurred. In depth information on coronary artery course and anatomy is not routinely collected in the audit dataset, and thus could not be included in the analysis. Detailed cost and treatment needs analyses could not be performed, as these data were not available.

## Conclusions

In a national cohort of TGA-IVS, mortality in the first 10 years of life was low (3%) and concentrated in the first year of life, while cardiac reintervention was higher, 10% at 10 years and associated with more days spent in hospital. Most patients will have a low overall hospital burden with a median of 1 outpatient day/year throughout childhood and adolescence. Use of BAS or age at ASO was not associated with adverse outcomes, but patient low-weight and critical clinical condition (requiring circulatory or renal support) were (Central Illustration).

**Central Illustration:**
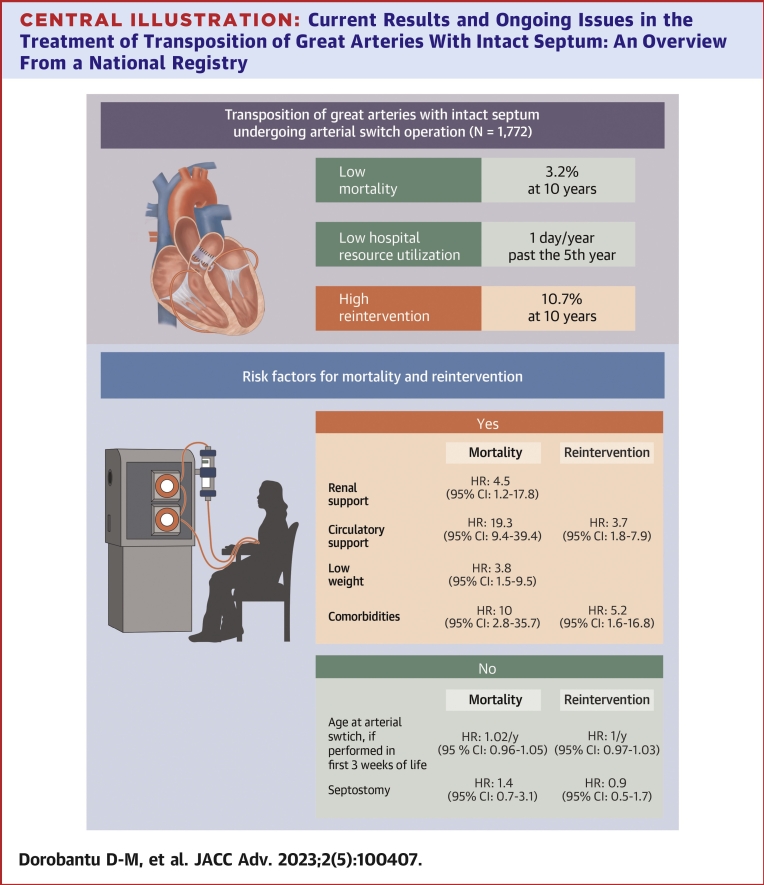
Current Results and Ongoing Issues in the Treatment of Transposition of Great Arteries With Intact Septum: An Overview From a National Registry

Our study shows that TGA-IVS treatment aimed at early ASO repair that led to favorable outcomes and allowed for some regional variability in practice in regard of timing of repair. Despite considerable variability in practice, the choice of first procedure, BAS or primary ASO, did not appear to impact outcomes, even when performed at similar ages, supporting primary early ASO as a feasible choice. The low average mortality and burden of hospitalization in this cohort is reassuring, but the relatively high reintervention rate and associated increase in hospital utilization reflect a subgroup where improvements in care are needed.

PERSPECTIVES **COMPETENCY IN MEDICAL KNOWLEDGE 1:** In TGA-IVS undergoing ASO, days spent in hospital decrease gradually from the first year of follow-up to a median of 1 day/year in adolescents, most being outpatient visits.

**COMPETENCY IN MEDICAL KNOWLEDGE 2:** Modern, guideline-oriented treatment choices achieve excellent outcomes in TGA-IVS. Risk factors for mortality and reintervention are related to severity of disease rather than treatment choices.

**TRANSLATIONAL OUTLOOK 1:** In a national cohort of simple TGA-IVS undergoing arterial switch at a median 9.5 days, markers of clinical severity and not age at arterial switch were associated with worse short- and long-term outcomes.

**TRANSLATIONAL OUTLOOK 2:** At similar age and weight, arterial switch as first procedure achieved similar short- and long-term outcomes compared to balloon atrial septostomy followed by arterial switch, including postoperative hospital resource utilization. Choosing one approach over the other should aim at limiting hypoxic time based on the clinical and logistical context.

## Funding support and author disclosures

This study is supported by the Health Foundation, an independent charity committed to bringing about better health and health care for people in the United Kingdom (Award number 685009). D.M. Dorobantu is supported by a PhD Studentship (grant MR/N0137941/1 for the GW4 BIOMED DTP), awarded to the Universities of Bath, Bristol, Cardiff, and Exeter from the 10.13039/501100000265Medical Research Council (MRC)/UKRI, unrelated to this work. The views expressed are those of the authors and not necessarily those of the National Health Service, National Institute for Health Research, or Department of Health. The Linking AUdit and National datasets in Congenital HEart Services (LAUNCHES) project received ethical approval from the Health Research Authority (reference: IRAS 246796) and the Confidentiality Advisory Group (reference: 18/CAG/0180). All other authors have reported that they have no relationships relevant to the contents of this paper to disclose.
