# Supplementary material for: Arterial Switch for Transposition of the Great Arteries: Treatment Timing, Late Outcomes, and Risk Factors
Source: JACC Adv. 2023 Jul 19;2(5):100407. doi: 10.1016/j.jacadv.2023.100407 (PMC11198700; doi:10.1016/j.jacadv.2023.100407)
Supplement: Supplemental information [file mmc1.docx]

**SUPPLEMENTAL APPENDIX**

**SUPPLEMENTAL METHODS**

## **Patient Selection and Classification**

As described in the main manuscript Figure 1, we selected patients born between 1 April 2000 and 31 March 2017, having a simple transposition of the great arteries with intact ventricular septum (TGA-IVS) treated with by arterial switch operation (ASO), preceeded or not by an initial balloon atrial septostomy (BAS), with at most other minor cardiac procedures in public hospitals from England or Wales done before the ASO. We restricted to residents in England or Wales to guarantee a complete intervention history using the LAUNCHES (Linking Audit and National datasets in Congenital Heart Services for Quality Improvement) dataset.

The automatic identification of TGA-IVS patients using the LAUNCHES dataset followed a three-step process. First, we included all patients having a TGA diagnosis or a ASO repair procedure code, as indicated by the diagnosis codes and specific procedure categories in Supplementary Table 1, both formats available from the National Congenital Heart Disease Audit (NCHDA).(1) Then, we excluded complex TGA patients having any record with a exclusion code from Supplementary Table 2, which uses the Partial Risk Adjustment in Surgery (PRAiS2) diagnosis categories,(2) and the NCHDA Specific Procedure categories for procedures. The identification of complex and VSD patients using those categories was limited, and we used the diagnosis and procedure codes in Supplementary Table 3 to refine the identification and exclusion of those patients. A further manual revision of those patients with no ASO resulted in the identification of 11 patients with initial septostomy who did not reach ASO, and the exclusion of 92 patients with not primary ASO or ambiguous treatment (details about these groups are provided in the manuscript).

## **Collected data and outcomes**

*Patient Characteristics*

We identified BAS procedures in NCHDA using the following procedure codes:

- 120141. Balloon atrial septostomy by pull back (Rashkind)
- 151066. Ineffective balloon atrial septostomy

Using the NCHDA ages at procedures, we could identify the BAS procedures performed before an ASO. The LAUNCHES data set contained additionally the financial year of procedures, estimated using year and month of birth and then age at procedure (i.e. with a 15-day uncertainty due to unavailable day of birth.(3) We anonymised the name of the treating hospital (twelve hospitals in total) available from NCHDA and when reporting numbers per centre we omitted two centres with small numbers (1 and 2 ASOs only).

We identified some patient characteristics at patient level (any entries in the LAUNCHES dataset). These were sex, available from NCHDA and Hospital Episode Statistics (HES), and preterm birth, antenatal diagnosis and congenital comorbidity (all three derived from NCHDA codes using PRAiS2 lookups).

We used NCHDA ages and weights at first intervention/BAS and at ASO. Using PRAiS2 lookups, we also extracted from NCHDA record indicators for perioperative acquired comorbidity, additional cardiac risk factors, severity of illness. ASO bypass time length (minutes) was available from NCHDA.

*Data availability periods*

Age at latest known life status was available from linkage to the Office of National Statistics (ONS), and otherwise the latest age in data set was used. Age at death was available from NCHDA (in-hospital mortality) and (mainly) from linkage to ONS mortality. Linkage to ONS was successful for most patients with a valid National Health System (NHS) number (England and Wales).

Procedural, intensive care and hospital utilisation data from LAUCNHES was covering different periods and therefore cohorts of analysis and available follow-up varied by data source. For all included TGA-IVS patients (born from April 2000 and residents in England or Wales), the LAUNCHES dataset allowed to know all their cardiac procedures as recorded in the NCHDA database. Data from the Paediatric Intensive Care Audit Network (PICANet) was available in LAUNCHES up to March 2017 for the majority of included patients born after October 2001, due to the terms of the PICANet Health Research Authority Confidentiality Group approval for processing identifiable information.(3) HES data from hospitals in England was available from birth up to March 2018 for inpatient admissions, from April 2003 up to March 2018 for outpatient visits (attended appointments completeness being excellent), and from April 2007 to March 2018 for A&E visits.

*Outcomes*

Other than treatment and patient related characteristics, we extracted the following ASO-related outcomes: mortality at 30 days from procedure, in hospital, and over the maximum possible follow-up period (limited by ONS mortality linkage); cardiac reintervention at 30 days, in hospital and over the maximum possible follow-up period (limited by NCHDA data availability); intensive care unit (ICU) utilisation, such as extracorporeal membrane oxygenation (ECMO), renal support, inotrope support, invasive and non-invasive ventilation; hospital and ICU lengths of stay (total, pre-ASO, and post-ASO); post-ASO hospital utilisation over the maximum possible follow-up (limited by linkage to HES availability), overall and then cardiac-related or non-cardiac, identified as explained next. Life status was ascertained using ONS mortality registry; when linkage to ONS was not available, their clinical records were used.

We adapted the approach in a previous LAUNCHES paper to identify cardiac contacts using the LAUNCHES data:(4)

1. Outpatient Cardiac Appointments were identified in HES data using the Treatment Speciality (TRETSPEF) field from HES OP (before 2004/05, TRETSPEF contained the consultant speciality instead of treatment speciality).
   1. Only the treatment specialities in Supplementary Table 4 were considered indicators of congenital heart disease (CHD) appointments.
   2. Patient attendance to the outpatient appointments was required (HES outpatient data also contained information, although likely incomplete, about appointments not attended or cancelled by provider).
2. Inpatient Cardiac Admissions were identified in HES data using the following Healthcare Resource Group (HRG) codes, noting different structural HRG versions during the period of study:
   1. E-codes (Cardiac surgery and primary cardiac conditions);
   2. HRG3 code : P25 (Cardiac conditions);
   3. HRG4 codes: PA22Z (Chest pain), PA23A and PA23B (Cardiac conditions with/without complications and comorbidities (CC)), and PA24Z (Arrhythmia or conduction disorders).
   4. HRG4+ codes: PE23A-PE23F (Paediatric cardiac conditions, with different CC scores), PE24A-PE24C (Paediatric arrhythmia or conduction disorders, with different CC scores), and PE62A-PE62C (Paediatric syncope and collapse, with different CC scores).
3. All NCHDA reported procedures were considered to be cardiac contacts.
4. A&E cardiac visits were identified in HES data using the HES Accidents and Emergency room (A&E) diagnosis code, which we required to be “20. Cardiac conditions”.

## **Statistical Analysis**

*Descriptive analysis of TGA-IVS patients and outcomes*

We described patient and treatment characteristics (Figure 1, Table 1 and main manuscript). We described variation over time and by centre in treatment (ASO as first procedure versus BAS followed by ASO) and in ages at ASO (Figure 2 and main manuscript). We described all outcomes enumerated in the previous section: short-term and perioperative outcomes were detailed in Table 2 and summarized in the main manuscript; post-ASO probabilities of mortality and reintervention were described in the main manuscript and Figure 3, with the underlying numbers provided in Supplementary Table 5 for mortality, in Supplementary Table 6 for (either surgical or transcatheter) cardiac reintervention, in Supplementary Table 7 for surgical reintervention and in Supplementary Table 8for transcatheter reintervention. In hospital resource utilization (overall, cardiac and other) was summarised in the main manuscript and Figure 5, with the numeric values for that figure provided in Supplementary Table 9 for overall hospital utilisation, Supplementary Table 10 for inpatient or outpatient stays, Supplementary Table 11 for inpatient only, Supplementary Table 12 for outpatient visits (no overlap with inpatient spells), Supplementary Table 13 for A&E visits only.

The different periods of availability for each data set meant that we had different follow-up times depending on outcome. We described only outcomes on years (of age) where we had patients covering the whole year. Post-ASO survival median (IQR) follow-up was 12.4 (8.6-16.6) years, with maximum value 21.8 years; post-ASO reintervention median (IQR) follow-up was 8.2 (4.0-12.7) years, with maximum value 16.9 years. We reported survival up to 21 years post-ASO and reintervention up to 16 years post-ASO. HES follow-up post-ASO spell had median (IQR) 8.8 (4.8-13.4) years, with maximum value 17.9 years. We reported hospital utilisation up to 17 years post-ASO spell.

The different periods of availability for each data set also meant that we had to account for right-censoring for all outcomes and additionally for left-truncation when describing hospital utilisation (pre-2009). Kaplan Meier (survival analysis) and conditional probability functions (competing risks analysis) were used to account for censoring when producing average and 95% CI estimates of survival or reintervention. When reporting days in hospital, we required patients to be alive and have data coverage on all needed data sets for at least part of the year reported. We added notes to Supplementary Tables from 9-13 clarifying which data sets were needed for each measure.

*Explore predictors for early, mid and late term outcomes, with a focus on the role of ASO timing, treatment type and impact of low weight*

In the main manuscript and Table 3 we presented our multivariable analysis of factors associated with early and late mortality and reintervention after ASO. We focused our analysis on the role of ASO timing, treatment type (use of BAS) and impact of low weight. Accordingly, we included in all models the variables age at ASO, BAS+ASO indicator (yes or primary ASO otherwise), and weight at ASO < 2.5Kg. We additionally performed univariable analyses for all fields in Table 1, testing the statistically significant fields into multivariable models. Having predetermined age, treatment, and low weight as predictors, there were few additional combinations of other variables being significant and in all those age at treatment and treatment type were not significant. We selected for display the same multivariable predictors at short and long term to allow for comparison, selecting the models with best pseudolikelihood and giving preference to fields available for the majority of patients.

*Investigate the differences in outcomes in TGA-IVS patients of comparable characteristics undergoing BAS or ASO as a first procedure*

We matched patients with BAS+ASO treatment (including those with only BAS followed by death) to patients undergoing only ASO, in a 1:1 ratio, using the following constraints:

- Their age at first procedure was at most 21.0 days (first three weeks of life), as recommended by current guidelines.(5)
- The age at first procedure had to be the same between matched patients (up to 1 day tolerance), to reflect both ASO and BAS were options for treatment at the same age
- The age-sex weight Z-score had to be the same between matched patients (up to 2 standard deviations tolerance), to account for preference on initial palliation over bypass surgery in low weight neonates
- The year of procedure had to be the same up to 3 years tolerance, to account for differences in choices by era

This resulted in 174 matches (348 patients). While ages at first repair were predominantly low for BAS treatments amongst the pooling sample of patients with first repair at first three weeks of age, the matching was dominated by the available ages at ASO in the primary ASO patients from the pooling sample (Supplementary Table 14). The age-sex weight z-scores of the matched sample were similarly distributed than the z-scores of the pooling sample except for the 5% and 95% extreme percentiles not appearing in the matched sample (Supplementary Table 15). The procedure years in the matched sample were balanced and similar to those in the pooling sample (Supplementary Table 16).

Mortality, reintervention and hospital resource utilization were compared between the two matched groups using a Pearson’s chi-squared test for independence, Welch’s t-test for difference of averages. In order to compare median days post-ASO spell in hospital per year, we built an augmented dataset with 17 years of follow-up and for each year the matched pairs of patients with follow-up data on that year, then run a quantile regression for median comparison by BAS+ASO vs primary ASO, adjusting for year of follow-up since ASO spell (parameterised using a cubic spline with 5 nodes to accommodate for a non-linear decreasing trend in days in hospital over years of follow-up); the p-value for the treatment type coefficient in the median regression was reported in Table 4.

**SUPPLEMENTAL TABLES**

## **Codes used in inclusion/exclusion**

| **Supplemental Table 1.** Diagnosis and Specific procedure codes used as first inclusion step to identify the simple TGA-IVS cohort. |
| --- |
| **NCHDA Code and Description (Inclusion)** |
| 010501. Discordant VA connections (TGA) |
| 010102. Transposition of great arteries (concordant AV & discordant VA connections) & IVS |
| **NCHDA specific procedure category used for Inclusion** |
| 13: transposition |
| AV, atrio-ventricular; TGA, transposition of the great arteries; TGA-IVS, TGA with intact septum; VA, ventriculo-arterial; |

| **Supplemental Table 2.** Primary Diagnosis and Specific Procedure categories from the NCHDA records in the LAUNCHES dataset used for exclusion of non-transposition of the great arteries with intact septum (TGA-IVS) patients. |
| --- |
| **Overall NCHDA diagnosis category used for Exclusion** |
| 1: Hypoplastic left heart syndrome |
| 2: Functionally univentricular heart (UVH) |
| 3: Common arterial trunk (truncus arteriosus) |
| 5: Interrupted Aortic Arch |
| 7: Pulmonary atresia & intact ventricular septum |
| 8: Pulmonary atresia & ventricular septal defect |
| 9: Miscellaneous primary congenital disease |
| 10: Atrioventricular septal defect |
| 11: Tetralogy of Fallot /Fallot-type double outlet right ventricle |
| 12: Aortic valve stenosis (isolated) |
| 15: Total anomalous pulmonary venous connection |
| 16: Aortic arch obstruction +/- ventricular septal defect +/- atrial septal defect |
| **NCHDA specific procedure category used for Exclusion** |
| 01: Norwood procedure |
| 04: Common arterial trunk (truncus arteriosus) and aorta arch repair |
| 05: Common arterial trunk (truncus arteriosus) repair |
| 06, 07: Double Switch or Rastelli-Senning repair of ccTGA a |
| 09: Rastelli or REV procedure |
| 10: Complex procedure for transposed great arteries |
| 11: Arterial switch and aorta arch repair |
| 12: Arterial switch and ventricular septal defect (VSD) repair |
| 14: Totally anomalous pulmonary venous connection (TAPVC) repair and arterial shunt |
| 15: Totally anomalous pulmonary venous connection (TAPVC) repair |
| 16: Fontan or Total Cavopulmonary Connection (TCPC) |
| 17: Glenn (Cavopulmonary (CP) shunt) |
| 19: Atrioventricular septal defect (AVSD) & Tetralogy of Fallot repair |
| 20: Complete atrioventricular septal defect (AVSD) repair |
| 21: Partial atrioventricular septal defect (AVSD) repair |
| 23, 24: Ross-Konno procedure |
| 25: Ross procedure (aortic valve-root replacement with pulmonary autograph) |
| 33: Pulmonary atresia & ventricular septal defect (VSD) repair |
| 34: Systemic-to-pulmonary collateral artery (MAPCA) unifocalisation procedure |
| 35: Tetralogy of Fallot with absent pulmonary valve repair |
| 36: Tetralogy of Fallot and Fallot-type double outlet right ventricle repair |
| 41: Aortopulmonary (AP) window repair |
| 42: Anomalous coronary artery repair |
| 43: Cor triatriatum (divided left atrium) repair |
| 46: Interrupted aortic arch repair |
| 47: Isolated coarctation/hypoplasia of aorta repair |
| 50: Closure of multiple ventricular septal defects (VSD) |
| 65: Stent placement at site of aortic coarctation |
| 66: Balloon dilation of native aortic coarctation-hypoplasia |
| 67: Balloon dilation of aortic re-coarctation |
| 68: Balloon dilation of aortic valve |
| LAUNCHES, Linking AUdit and National datasets in Congenital HEart Services for Quality Improvement; NCHDA, National Congenital Heart Disease Audit. |

| **Supplemental Table 3.** Diagnosis and procedure codes as third exclusion step to identify the simple transposition of the great arteries with intact septum (TGA-IVS) cohort. | |
| --- | --- |
| **NCHDA Code** | **NCHDA Code Description** |
| 010103 | Congenitally corrected transposition of great arteries (discordant atrio-ventricular(AV) & ventriculo-arterial(VA) connections) |
| 010104 | Double outlet right ventricle |
| 010106 | Pulmonary atresia + ventricular septal defect (VSD) (including Fallot type) |
| 010117 | Double outlet right ventricle: Fallot type (subaortic or doubly committed VSD & pulmonary stenosis) |
| 010118 | Double outlet right ventricle: transposition type (subpulmonary VSD) |
| 010119 | Double outlet right ventricle: with non-committed VSD |
| 010125 | Pulmonary atresia + VSD + systemic-to-pulmonary collateral artery(ies) (MAPCA(s)) |
| 010140 | Double outlet right ventricle: subaortic or doubly committed VSD without pulmonary stenosis ('VSD type') |
| 010503 | Double outlet left ventricle |
| 030104 | Right isomerism (‘asplenia’) |
| 030105 | Left isomerism (‘polysplenia’) |
| 040806 | Obstructed pulmonary venous connection(s) |
| 040820 | Totally anomalous pulmonary venous connection: infracardiac |
| 060506 | Atrioventricular septal defect (AVSD) AV valvar regurgitation |
| 060600 | Atrioventricular septal defect |
| 060601 | AVSD: isolated atrial component (primum atrial septal defect (ASD))(partial) |
| 060608 | AVSD: isolated ventricular component |
| 060609 | AVSD: atrial & ventricular components with common AV orifice (complete) |
| 060610 | AVSD: atrial & (restrictive) ventricular components + separate AV valves ('intermediate') |
| 071000 | VSD |
| 071001 | Perimembranous VSD |
| 071012 | VSD + malaligned outlet septum |
| 071200 | Subarterial VSD |
| 071201 | Doubly committed subarterial VSD |
| 071405 | Inlet VSD |
| 071501 | Tiny VSD (Maladie de Roger) |
| 071504 | Multiple VSDs |
| 071505 | Single VSD |
| 152202 | Residual VSD |
| 072000 | Ventricular septal abnormality |
| 092931 | Interrupted aortic arch |
| 120000 | Totally anomalous pulmonary venous connection repair |
| 120801 | VSD closure |
| 121141 | Truncal valve replacement |
| 123037 | Fontan type procedure revision or conversion |
| 123054 | Total cavopulmonary connection (TCPC) using extracardiac inferior caval vein (IVC)-pulmonary artery conduit |
| 151013 | Obstruction of right atrial conduit (TCPC) |
| NCHDA, National Congenital Heart Disease Audit. | |

## **Codes used in classification of hospital episodes**

| **Supplemental Table 4.** Treatment specialities (code and description) considered as indicative of cardiac contacts in Hospital Episodes Statistics outpatient records.[REF transitions] | |
| --- | --- |
| **Treatment Speciality Code** | **Treatment Speciality Description** |
| 170 | Cardiothoracic Surgery (where there are no separate services for cardiac and thoracic surgery) |
| 172 | Cardiac Surgery |
| 174 | Cardiothoracic Transplantation (recognised specialist services only - includes 'outreach' facilities) |
| 221 | Paediatric Cardiac Surgery (From 2006-07) |
| 320 | Cardiology |
| 321 | Paediatric Cardiology |
| 331 | Congenital Heart Disease Service (From April 2013) |

## **Arterial switch operation center case load**

| **Supplemental Table 5.** Arterial switch operation (ASO) case load by era and center between 2000-2016 in England and Wales | | | | | | | | | | |
| --- | --- | --- | --- | --- | --- | --- | --- | --- | --- | --- |
|  | **Total number of ASO** | | | | | **Average ASO/year** | | | | |
|  | **2000-2004** | **2005-2008** | **2009-2012** | **2013-2016** | **All years** | **2000-2004** | **2005-2008** | **2009-2012** | **2013-2016** | **All years** |
| H1 | 84 | 68 | 69 | 55 | 276 | 16 | 17 | 17 | 17 | 14 |
| H2 | 57 | 54 | 60 | 52 | 223 | 13 | 11 | 14 | 15 | 13 |
| H3 | 69 | 43 | 46 | 42 | 200 | 12 | 14 | 11 | 12 | 11 |
| H4 | 54 | 50 | 51 | 39 | 194 | 11 | 11 | 13 | 13 | 10 |
| H5 | 58 | 46 | 52 | 38 | 194 | 11 | 12 | 12 | 13 | 10 |
| H6 | 51 | 37 | 40 | 46 | 174 | 10 | 10 | 9 | 10 | 12 |
| H7 | 49 | 41 | 38 | 34 | 162 | 10 | 10 | 10 | 10 | 9 |
| H8 | 28 | 33 | 30 | 29 | 120 | 7 | 6 | 8 | 8 | 7 |
| H9 | 33 | 19 | 31 | 33 | 116 | 7 | 7 | 5 | 8 | 8 |
| H10 | 41 | 21 | 23 | 25 | 110 | 6 | 8 | 5 | 6 | 6 |
| Total^a^ | 527 | 412 | 440 | 393 | 1,772 | 104 | 105 | 103 | 110 | 98 |
| ^a^Two centers not shown having n=2 and n=1 total ASO  H1 - H10 – centers performing ASO, in order of size. | | | | | | | | | | |

##

## **Detailed long term outcomes**

| **Supplemental Table 6.** Probability of death (Kaplan-Meier) over 21 years post-arterial switch operation (ASO) (maximum follow-up was 21.8 years). | | | | |
| --- | --- | --- | --- | --- |
| **Time from ASO (months)** | **At risk** | **Died** | **Censored** | **Probability of death** |
| 0 | 1772 |  |  |  |
| 1 | 1741 | 31 | 0 | 1.7% (1.2%-2.5%) |
| 2 | 1729 | 12 | 0 | 2.4% (1.8%-3.3%) |
| 3 | 1725 | 3 | 1 | 2.6% (2.0%-3.5%) |
| 4 | 1724 | 1 | 0 | 2.7% (2.0%-3.5%) |
| 5 | 1722 | 2 | 0 | 2.8% (2.1%-3.6%) |
| 6 | 1722 | 0 | 0 | 2.8% (2.1%-3.6%) |
| 7 | 1722 | 0 | 0 | 2.8% (2.1%-3.6%) |
| 8 | 1722 | 0 | 0 | 2.8% (2.1%-3.6%) |
| 9 | 1722 | 0 | 0 | 2.8% (2.1%-3.6%) |
| 10 | 1722 | 0 | 0 | 2.8% (2.1%-3.6%) |
| 11 | 1722 | 0 | 0 | 2.8% (2.1%-3.6%) |
| 12 | 1721 | 1 | 0 | 2.8% (2.1%-3.7%) |
| **Time from ASO (years)** | **At risk** | **Died** | **Censored** | **Probability of death** |
| 0 | 1772 |  |  |  |
| 1 | 1721 | 50 | 1 | 2.8% (2.1%-3.7%) |
| 2 | 1712 | 2 | 7 | 2.9% (2.2%-3.8%) |
| 3 | 1711 | 1 | 0 | 3.0% (2.3%-3.9%) |
| 4 | 1705 | 2 | 4 | 3.1% (2.4%-4.0%) |
| 5 | 1682 | 1 | 22 | 3.2% (2.4%-4.1%) |
| 6 | 1582 | 0 | 100 | 3.2% (2.4%-4.1%) |
| 7 | 1483 | 0 | 99 | 3.2% (2.4%-4.1%) |
| 8 | 1398 | 1 | 84 | 3.2% (2.5%-4.2%) |
| 9 | 1274 | 0 | 124 | 3.2% (2.5%-4.2%) |
| 10 | 1182 | 0 | 92 | 3.2% (2.5%-4.2%) |
| 11 | 1061 | 0 | 121 | 3.2% (2.5%-4.2%) |
| 12 | 949 | 0 | 112 | 3.2% (2.5%-4.2%) |
| 13 | 832 | 0 | 117 | 3.2% (2.5%-4.2%) |
| 14 | 720 | 0 | 112 | 3.2% (2.5%-4.2%) |
| 15 | 607 | 0 | 113 | 3.2% (2.5%-4.2%) |
| 16 | 506 | 0 | 101 | 3.2% (2.5%-4.2%) |
| 17 | 414 | 0 | 92 | 3.2% (2.5%-4.2%) |
| 18 | 326 | 0 | 88 | 3.2% (2.5%-4.2%) |
| 19 | 238 | 0 | 88 | 3.2% (2.5%-4.2%) |
| 20 | 145 | 0 | 93 | 3.2% (2.5%-4.2%) |
| 21 | 63 | 0 | 82 | 3.2% (2.5%-4.2%) |
| For each time point, the table contains the number at risk at that time (did not die and were not lost due to data censoring), the number that died and the number lost to data censoring between the previous time and the current time, and the estimated average and 95% CI for the risk of death from ASO to the current time point. For example, at one month, there were still 1741 patients at risk, there had been 31 deaths and no patients lost to censoring; therefore, the estimated risk of death was 1.7% (= 31/1772).  30-day mortality was slightly lower than 1-month mortality: 30 patients out of 1,772 died at 30 days (all patients had enough data), the average (95% CI) estimate being 1.7% (1.2%-2.4%). | | | | |

| **Supplemental Table 7.** Probability of cardiac reintervention conditional on survival (Conditional Probability Function - CPF) and cumulative cardiac reintervention (Cumulative Incidence Function - CIF) over 16 years of follow-up (maximum follow-up was 16.9 years). | | | | | | | |
| --- | --- | --- | --- | --- | --- | --- | --- |
| **Time (months)** | **At risk (no reintervention and alive)** | **Cardiac reintervention (cumulative)** | **Died without reintervention (cumulative)** | **Not enough data (cumulative)** | **CIF cardiac reintervention** | **CIF death without reintervention** | **CPF cardiac reintervention if alive** |
| 0 | 1772 |  |  |  |  |  |  |
| 1 | 1704 (96.2%) | 34 (1.9%) | 23 (1.3%) | 11 (0.6%) | 1.9% (1.4%-2.6%) | 1.3% (0.9%-1.9%) | 1.9% (1.3%-2.6%) |
| 2 | 1683 (95.0%) | 42 (2.4%) | 33 (1.9%) | 14 (0.8%) | 2.4% (1.7%-3.2%) | 1.9% (1.3%-2.6%) | 2.4% (1.7%-3.1%) |
| 3 | 1660 (93.7%) | 48 (2.7%) | 36 (2.0%) | 28 (1.6%) | 2.7% (2.0%-3.6%) | 2.0% (1.5%-2.8%) | 2.8% (2.0%-3.6%) |
| 4 | 1641 (92.6%) | 58 (3.3%) | 37 (2.1%) | 36 (2.0%) | 3.3% (2.5%-4.2%) | 2.1% (1.5%-2.9%) | 3.4% (2.5%-4.2%) |
| 5 | 1624 (91.6%) | 68 (3.8%) | 38 (2.1%) | 42 (2.4%) | 3.9% (3.0%-4.9%) | 2.2% (1.6%-2.9%) | 4.0% (3.0%-4.9%) |
| 6 | 1603 (90.5%) | 81 (4.6%) | 38 (2.1%) | 50 (2.8%) | 4.6% (3.7%-5.7%) | 2.2% (1.6%-2.9%) | 4.7% (3.7%-5.7%) |
| 7 | 1586 (89.5%) | 89 (5.0%) | 38 (2.1%) | 59 (3.3%) | 5.1% (4.1%-6.2%) | 2.2% (1.6%-2.9%) | 5.2% (4.2%-6.3%) |
| 8 | 1572 (88.7%) | 98 (5.5%) | 38 (2.1%) | 64 (3.6%) | 5.6% (4.6%-6.8%) | 2.2% (1.6%-2.9%) | 5.7% (4.6%-6.9%) |
| 9 | 1560 (88.0%) | 105 (5.9%) | 38 (2.1%) | 69 (3.9%) | 6.0% (5.0%-7.2%) | 2.2% (1.6%-2.9%) | 6.2% (5.0%-7.3%) |
| 10 | 1548 (87.4%) | 113 (6.4%) | 38 (2.1%) | 73 (4.1%) | 6.5% (5.4%-7.7%) | 2.2% (1.6%-2.9%) | 6.7% (5.5%-7.8%) |
| 11 | 1536 (86.7%) | 116 (6.5%) | 38 (2.1%) | 82 (4.6%) | 6.7% (5.6%-7.9%) | 2.2% (1.6%-2.9%) | 6.8% (5.6%-8.0%) |
| **Time (years)** | **At risk (no reintervention and alive)** | **Cardiac reintervention (cumulative)** | **Died without reintervention (cumulative)** | **Not enough data (cumulative)** | **CIF cardiac reintervention** | **CIF death without reintervention** | **CPF cardiac reintervention if alive** |
| 0 | 1772 |  |  |  |  |  |  |
| 1 | 1519 (85.7%) | 122 (6.9%) | 39 (2.2%) | 92 (5.2%) | 7% (5.9%-8.3%) | 2.2% (1.6%-3.0%) | 7.2% (6.0%-8.4%) |
| 2 | 1404 (79.2%) | 143 (8.1%) | 40 (2.3%) | 185 (10.4%) | 8.3% (7.1%-9.7%) | 2.3% (1.7%-3.1%) | 8.5% (7.2%-9.9%) |
| 3 | 1334 (75.3%) | 149 (8.4%) | 41 (2.3%) | 248 (14.0%) | 8.7% (7.4%-10.1%) | 2.3% (1.7%-3.1%) | 8.9% (7.6%-10.3%) |
| 4 | 1225 (69.1%) | 153 (8.6%) | 43 (2.4%) | 351 (19.8%) | 9% (7.7%-10.4%) | 2.5% (1.8%-3.3%) | 9.2% (7.8%-10.6%) |
| 5 | 1134 (64.0%) | 163 (9.2%) | 44 (2.5%) | 431 (24.3%) | 9.7% (8.4%-11.2%) | 2.5% (1.8%-3.3%) | 10.0% (8.5%-11.5%) |
| 6 | 1031 (58.2%) | 165 (9.3%) | 44 (2.5%) | 532 (30.0%) | 9.9% (8.5%-11.4%) | 2.6% (1.9%-3.4%) | 10.2% (8.7%-11.6%) |
| 7 | 933 (52.7%) | 165 (9.3%) | 44 (2.5%) | 630 (35.6%) | 9.9% (8.5%-11.4%) | 2.6% (1.9%-3.4%) | 10.2% (8.7%-11.6%) |
| 8 | 824 (46.5%) | 166 (9.4%) | 44 (2.5%) | 738 (41.6%) | 10.0% (8.6%-11.5%) | 2.6% (1.9%-3.4%) | 10.3% (8.8%-11.8%) |
| 9 | 712 (40.2%) | 166 (9.4%) | 44 (2.5%) | 850 (48.0%) | 10.0% (8.6%-11.5%) | 2.6% (1.9%-3.4%) | 10.3% (8.8%-11.8%) |
| 10 | 617 (34.8%) | 169 (9.5%) | 44 (2.5%) | 942 (53.2%) | 10.4% (8.9%-12.0%) | 2.6% (1.9%-3.4%) | 10.7% (9.1%-12.2%) |
| 11 | 528 (29.8%) | 172 (9.7%) | 44 (2.5%) | 1028 (58.0%) | 10.8% (9.3%-12.5%) | 2.6% (1.9%-3.4%) | 11.1% (9.5%-12.7%) |
| 12 | 446 (25.2%) | 174 (9.8%) | 44 (2.5%) | 1108 (62.5%) | 11.2% (9.6%-12.9%) | 2.6% (1.9%-3.4%) | 11.5% (9.8%-13.2%) |
| 13 | 363 (20.5%) | 180 (10.2%) | 44 (2.5%) | 1185 (66.9%) | 12.5% (10.6%-14.5%) | 2.6% (1.9%-3.4%) | 12.8% (10.8%-14.8%) |
| 14 | 273 (15.4%) | 183 (10.3%) | 44 (2.5%) | 1272 (71.8%) | 13.2% (11.2%-15.4%) | 2.6% (1.9%-3.4%) | 13.6% (11.4%-15.7%) |
| 15 | 175 (9.9%) | 183 (10.3%) | 44 (2.5%) | 1370 (77.3%) | 13.2% (11.2%-15.4%) | 2.6% (1.9%-3.4%) | 13.6% (11.4%-15.7%) |
| 16 | 85 (4.8%) | 183 (10.3%) | 44 (2.5%) | 1460 (82.4%) | 13.2% (11.2%-15.4%) | 2.6% (1.9%-3.4%) | 13.6% (11.4%-15.7%) |
| For each time point, the table contains the number left at risk at that time (did not have a reintervention, did not die and were not lost due to data censoring), the number that died and the number lost to data censoring between the previous time and the current time, and the estimated average and 95% CI for cumulative incidence functions (CIF) of reintervention and of death without reintervention (competing risk) and for the conditional probability function of reintervention if alive (CPF). | | | | | | | |

| **Supplemental Table 8.** Probability of cardiac surgical reintervention conditional on survival (Conditional Probability Function - CPF) and cumulative cardiac reintervention (Cumulative Incidence Function - CIF) over 16 years of follow-up (maximum follow-up was 16.9 years). | | | | | | | |
| --- | --- | --- | --- | --- | --- | --- | --- |
| **Time (months)** | **At risk (no reintervention and alive)** | **Surgical reintervention (cumulative)** | **Died without reintervention (cumulative)** | **Not enough data (cumulative)** | **CIF surgical reintervention** | **CIF death without reintervention** | **CPF surgical reintervention if alive** |
| 0 | 1772 |  |  |  |  |  |  |
| 1 | 1715 (96.8%) | 22 (1.2%) | 24 (1.4%) | 11 (0.6%) | 1.2% (0.8%-1.8%) | 1.4% (0.9%-2.0%) | 1.3% (0.7%-1.8%) |
| 2 | 1695 (95.7%) | 28 (1.6%) | 35 (2.0%) | 14 (0.8%) | 1.6% (1.1%-2.3%) | 2.0% (1.4%-2.7%) | 1.6% (1.0%-2.2%) |
| 3 | 1676 (94.6%) | 30 (1.7%) | 38 (2.1%) | 28 (1.6%) | 1.7% (1.2%-2.4%) | 2.2% (1.6%-2.9%) | 1.7% (1.1%-2.4%) |
| 4 | 1665 (94.0%) | 32 (1.8%) | 39 (2.2%) | 36 (2.0%) | 1.8% (1.3%-2.5%) | 2.2% (1.6%-3.0%) | 1.9% (1.2%-2.5%) |
| 5 | 1654 (93.3%) | 36 (2.0%) | 40 (2.3%) | 42 (2.4%) | 2.0% (1.5%-2.8%) | 2.3% (1.6%-3.0%) | 2.1% (1.4%-2.8%) |
| 6 | 1640 (92.6%) | 42 (2.4%) | 40 (2.3%) | 50 (2.8%) | 2.4% (1.8%-3.2%) | 2.3% (1.6%-3.0%) | 2.5% (1.7%-3.2%) |
| 7 | 1629 (91.9%) | 44 (2.5%) | 40 (2.3%) | 59 (3.3%) | 2.5% (1.9%-3.3%) | 2.3% (1.6%-3.0%) | 2.6% (1.8%-3.3%) |
| 8 | 1622 (91.5%) | 46 (2.6%) | 40 (2.3%) | 64 (3.6%) | 2.6% (2.0%-3.5%) | 2.3% (1.6%-3.0%) | 2.7% (1.9%-3.5%) |
| 9 | 1614 (91.1%) | 49 (2.8%) | 40 (2.3%) | 69 (3.9%) | 2.8% (2.1%-3.7%) | 2.3% (1.6%-3.0%) | 2.9% (2.1%-3.7%) |
| 10 | 1608 (90.7%) | 51 (2.9%) | 40 (2.3%) | 73 (4.1%) | 2.9% (2.2%-3.8%) | 2.3% (1.6%-3.0%) | 3.0% (2.2%-3.8%) |
| 11 | 1595 (90.0%) | 54 (3.0%) | 40 (2.3%) | 83 (4.7%) | 3.1% (2.4%-4.0%) | 2.3% (1.6%-3.0%) | 3.2% (2.3%-4.0%) |
| **Time (years)** | **At risk (no reintervention and alive)** | **Surgical reintervention (cumulative)** | **Died without reintervention (cumulative)** | **Not enough data (cumulative)** | **CIF surgical reintervention** | **CIF death without reintervention** | **CPF surgical reintervention if alive** |
| 0 | 1772 |  |  |  |  |  |  |
| 1 | 1580 (89.2%) | 58 (3.3%) | 41 (2.3%) | 93 (5.2%) | 3.3% (2.6%-4.3%) | 2.3% (1.7%-3.1%) | 3.4% (2.6%-4.3%) |
| 2 | 1472 (83.1%) | 70 (4.0%) | 42 (2.4%) | 188 (10.6%) | 4.1% (3.2%-5.1%) | 2.4% (1.8%-3.2%) | 4.2% (3.2%-5.1%) |
| 3 | 1403 (79.2%) | 72 (4.1%) | 43 (2.4%) | 254 (14.3%) | 4.2% (3.3%-5.2%) | 2.5% (1.8%-3.3%) | 4.3% (3.3%-5.3%) |
| 4 | 1291 (72.9%) | 72 (4.1%) | 45 (2.5%) | 364 (20.5%) | 4.2% (3.3%-5.2%) | 2.6% (1.9%-3.4%) | 4.3% (3.3%-5.3%) |
| 5 | 1194 (67.4%) | 79 (4.5%) | 46 (2.6%) | 453 (25.6%) | 4.7% (3.8%-5.8%) | 2.6% (1.9%-3.4%) | 4.9% (3.8%-5.9%) |
| 6 | 1087 (61.3%) | 80 (4.5%) | 46 (2.6%) | 559 (31.5%) | 4.8% (3.9%-5.9%) | 2.7% (2.0%-3.5%) | 5.0% (3.9%-6.0%) |
| 7 | 987 (55.7%) | 80 (4.5%) | 46 (2.6%) | 659 (37.2%) | 4.8% (3.9%-5.9%) | 2.7% (2.0%-3.5%) | 5.0% (3.9%-6.0%) |
| 8 | 870 (49.1%) | 82 (4.6%) | 47 (2.7%) | 773 (43.6%) | 5.0% (4.0%-6.2%) | 2.8% (2.1%-3.7%) | 5.2% (4.1%-6.3%) |
| 9 | 756 (42.7%) | 83 (4.7%) | 47 (2.7%) | 886 (50.0%) | 5.1% (4.1%-6.3%) | 2.8% (2.1%-3.7%) | 5.3% (4.2%-6.4%) |
| 10 | 661 (37.3%) | 85 (4.8%) | 47 (2.7%) | 979 (55.2%) | 5.4% (4.3%-6.6%) | 2.8% (2.1%-3.7%) | 5.5% (4.4%-6.7%) |
| 11 | 566 (31.9%) | 88 (5.0%) | 47 (2.7%) | 1071 (60.4%) | 5.8% (4.7%-7.2%) | 2.8% (2.1%-3.7%) | 6.0% (4.7%-7.3%) |
| 12 | 479 (27.0%) | 91 (5.1%) | 47 (2.7%) | 1155 (65.2%) | 6.4% (5.1%-7.8%) | 2.8% (2.1%-3.7%) | 6.5% (5.1%-8.0%) |
| 13 | 391 (22.1%) | 92 (5.2%) | 47 (2.7%) | 1242 (70.1%) | 6.6% (5.2%-8.1%) | 2.8% (2.1%-3.7%) | 6.8% (5.3%-8.2%) |
| 14 | 298 (16.8%) | 93 (5.2%) | 47 (2.7%) | 1334 (75.3%) | 6.8% (5.4%-8.4%) | 2.8% (2.1%-3.7%) | 7.0% (5.5%-8.5%) |
| 15 | 190 (10.7%) | 93 (5.2%) | 47 (2.7%) | 1442 (81.4%) | 6.8% (5.4%-8.4%) | 2.8% (2.1%-3.7%) | 7.0% (5.5%-8.5%) |
| 16 | 94 (5.3%) | 93 (5.2%) | 47 (2.7%) | 1538 (86.8%) | 6.8% (5.4%-8.4%) | 2.8% (2.1%-3.7%) | 7.0% (5.5%-8.5%) |
| For each time point, the table contains the number left at risk at that time (did not have a reintervention, did not die and were not lost due to data censoring), the number that died and the number lost to data censoring between the previous time and the current time, and the estimated average and 95% CI for cumulative incidence functions (CIF) of reintervention and of death without reintervention (competing risk) and for the conditional probability function of reintervention if alive (CPF). | | | | | | | |

| **Supplemental Table 9.** Probability of cardiac transcatheter reintervention conditional on survival (Conditional Probability Function - CPF) and cumulative cardiac reintervention (Cumulative Incidence Function - CIF) over 16 years of follow-up (maximum follow-up was 16.9 years). | | | | | | | |
| --- | --- | --- | --- | --- | --- | --- | --- |
| **Time (months)** | **At risk (no reintervention and alive)** | **Transcatheter reintervention (cumulative)** | **Died without reintervention (cumulative)** | **Not enough data (cumulative)** | **CIF transcatheter reintervention** | **CIF death without reintervention** | **CPF transcatheter reintervention if alive** |
| 0 | 1772 |  |  |  |  |  |  |
| 1 | 1719 (97.0%) | 12 (0.7%) | 30 (1.7%) | 11 (0.6%) | 0.7% (0.4%-1.2%) | 1.7% (1.2%-2.4%) | 0.7% (0.3%-1.1%) |
| 2 | 1702 (96.0%) | 14 (0.8%) | 41 (2.3%) | 15 (0.8%) | 0.8% (0.5%-1.3%) | 2.3% (1.7%-3.1%) | 0.8% (0.4%-1.2%) |
| 3 | 1681 (94.9%) | 18 (1.0%) | 44 (2.5%) | 29 (1.6%) | 1.0% (0.6%-1.6%) | 2.5% (1.8%-3.3%) | 1.0% (0.6%-1.5%) |
| 4 | 1662 (93.8%) | 28 (1.6%) | 45 (2.5%) | 37 (2.1%) | 1.6% (1.1%-2.3%) | 2.6% (1.9%-3.4%) | 1.6% (1.0%-2.2%) |
| 5 | 1646 (92.9%) | 36 (2.0%) | 47 (2.7%) | 43 (2.4%) | 2.1% (1.5%-2.8%) | 2.7% (2.0%-3.5%) | 2.1% (1.4%-2.8%) |
| 6 | 1630 (92.0%) | 44 (2.5%) | 47 (2.7%) | 51 (2.9%) | 2.5% (1.9%-3.3%) | 2.7% (2.0%-3.5%) | 2.6% (1.8%-3.4%) |
| 7 | 1615 (91.1%) | 50 (2.8%) | 47 (2.7%) | 60 (3.4%) | 2.9% (2.2%-3.7%) | 2.7% (2.0%-3.5%) | 3.0% (2.1%-3.8%) |
| 8 | 1601 (90.3%) | 59 (3.3%) | 47 (2.7%) | 65 (3.7%) | 3.4% (2.6%-4.3%) | 2.7% (2.0%-3.5%) | 3.5% (2.6%-4.4%) |
| 9 | 1587 (89.6%) | 67 (3.8%) | 47 (2.7%) | 71 (4.0%) | 3.9% (3.0%-4.9%) | 2.7% (2.0%-3.5%) | 4.0% (3.0%-4.9%) |
| 10 | 1576 (88.9%) | 74 (4.2%) | 47 (2.7%) | 75 (4.2%) | 4.3% (3.4%-5.3%) | 2.7% (2.0%-3.5%) | 4.4% (3.4%-5.4%) |
| 11 | 1564 (88.3%) | 76 (4.3%) | 47 (2.7%) | 85 (4.8%) | 4.4% (3.5%-5.4%) | 2.7% (2.0%-3.5%) | 4.5% (3.5%-5.5%) |
| **Time (years)** | **At risk (no reintervention and alive)** | **Transcatheter reintervention (cumulative)** | **Died without reintervention (cumulative)** | **Not enough data (cumulative)** | **CIF transcatheter reintervention** | **CIF death without reintervention** | **CPF transcatheter reintervention if alive** |
| 0 | 1772 |  |  |  |  |  |  |
| 1 | 1550 (87.5%) | 79 (4.5%) | 48 (2.7%) | 95 (5.4%) | 4.6% (3.7%-5.6%) | 2.7% (2.0%-3.6%) | 4.7% (3.7%-5.7%) |
| 2 | 1437 (81.1%) | 94 (5.3%) | 50 (2.8%) | 191 (10.8%) | 5.5% (4.5%-6.6%) | 2.9% (2.1%-3.7%) | 5.7% (4.5%-6.8%) |
| 3 | 1361 (76.8%) | 100 (5.6%) | 51 (2.9%) | 260 (14.7%) | 5.9% (4.8%-7.1%) | 2.9% (2.2%-3.8%) | 6.1% (4.9%-7.2%) |
| 4 | 1248 (70.4%) | 105 (5.9%) | 53 (3.0%) | 366 (20.7%) | 6.2% (5.2%-7.5%) | 3.1% (2.3%-3.9%) | 6.4% (5.2%-7.6%) |
| 5 | 1161 (65.5%) | 110 (6.2%) | 54 (3.0%) | 447 (25.2%) | 6.6% (5.5%-7.9%) | 3.1% (2.3%-3.9%) | 6.8% (5.6%-8.1%) |
| 6 | 1053 (59.4%) | 113 (6.4%) | 54 (3.0%) | 552 (31.2%) | 6.9% (5.7%-8.2%) | 3.1% (2.4%-4.0%) | 7.1% (5.8%-8.3%) |
| 7 | 954 (53.8%) | 113 (6.4%) | 54 (3.0%) | 651 (36.7%) | 6.9% (5.7%-8.2%) | 3.1% (2.4%-4.0%) | 7.1% (5.8%-8.3%) |
| 8 | 841 (47.5%) | 114 (6.4%) | 54 (3.0%) | 763 (43.1%) | 7.0% (5.8%-8.3%) | 3.1% (2.4%-4.0%) | 7.2% (5.9%-8.5%) |
| 9 | 727 (41.0%) | 114 (6.4%) | 54 (3.0%) | 877 (49.5%) | 7.0% (5.8%-8.3%) | 3.1% (2.4%-4.0%) | 7.2% (5.9%-8.5%) |
| 10 | 630 (35.6%) | 116 (6.5%) | 54 (3.0%) | 972 (54.9%) | 7.2% (6.0%-8.6%) | 3.1% (2.4%-4.0%) | 7.5% (6.1%-8.8%) |
| 11 | 540 (30.5%) | 118 (6.7%) | 54 (3.0%) | 1060 (59.8%) | 7.5% (6.2%-8.9%) | 3.1% (2.4%-4.0%) | 7.8% (6.4%-9.2%) |
| 12 | 458 (25.8%) | 118 (6.7%) | 54 (3.0%) | 1142 (64.4%) | 7.5% (6.2%-8.9%) | 3.1% (2.4%-4.0%) | 7.8% (6.4%-9.2%) |
| 13 | 372 (21.0%) | 123 (6.9%) | 54 (3.0%) | 1223 (69.0%) | 8.6% (7.1%-10.3%) | 3.1% (2.4%-4.0%) | 8.9% (7.2%-10.6%) |
| 14 | 280 (15.8%) | 125 (7.1%) | 54 (3.0%) | 1313 (74.1%) | 9.1% (7.5%-11.0%) | 3.1% (2.4%-4.0%) | 9.4% (7.6%-11.3%) |
| 15 | 178 (10.0%) | 125 (7.1%) | 54 (3.0%) | 1415 (79.9%) | 9.1% (7.5%-11.0%) | 3.1% (2.4%-4.0%) | 9.4% (7.6%-11.3%) |
| 16 | 88 (5.0%) | 125 (7.1%) | 54 (3.0%) | 1505 (84.9%) | 9.1% (7.5%-11.0%) | 3.1% (2.4%-4.0%) | 9.4% (7.6%-11.3%) |
| For each time point, the table contains the number left at risk at that time (did not have a reintervention, did not die and were not lost due to data censoring), the number that died and the number lost to data censoring between the previous time and the current time, and the estimated average and 95% CI for cumulative incidence functions (CIF) of reintervention and of death without reintervention (competing risk) and for the conditional probability function of reintervention if alive (CPF). | | | | | | | |

## **Detailed hospital resource utilization**

| **Supplemental Table 10.** Number of days spent at hospital (as either inpatient, outpatient or Accidents and Emergency room) by years from arterial switch operation (ASO) spell discharge. | | | | | | | | | | |
| --- | --- | --- | --- | --- | --- | --- | --- | --- | --- | --- |
| **Years from ASO spell** | **Total time in hospital (days)** | | | | | | | | | **Patient number** |
|  | *mean* | | | *median (IQR)* | | | *range* | | |  |
|  | **overall** | **cardiac** | **other** | **overall** | **cardiac** | **other** | **overall** | **cardiac** | **other** |  |
| 1^st^ year | 9.5 | 4.6 | 4.8 | 7 (4-10) | 3 (2-5) | 3 (1-6) | (0-264) | (0-259) | (0-182) | 1,078 |
| 2^nd^ year | 3.7 | 1.2 | 2.5 | 2 (1-4) | 1 (1-1) | 1 (0-3) | (0-121) | (0-63) | (0-67) | 1,142 |
| 3^rd^ year | 2.9 | 0.9 | 2.0 | 2 (1-3) | 1 (0-1) | 1 (0-2) | (0-87) | (0-23) | (0-64) | 1,131 |
| 4^th^ year | 2.6 | 0.8 | 1.8 | 1 (1-3) | 1 (0-1) | 1 (0-2) | (0-72) | (0-27) | (0-66) | 1,134 |
| 5^th^ year | 2.6 | 1.0 | 1.7 | 1 (1-3) | 1 (0-1) | 0 (0-2) | (0-197) | (0-194) | (0-34) | 1,154 |
| 6^th^ year | 2.4 | 0.7 | 1.7 | 1 (1-3) | 1 (0-1) | 0 (0-2) | (0-66) | (0-32) | (0-38) | 1,120 |
| 7^th^ year | 2.1 | 0.7 | 1.5 | 1 (0-3) | 1 (0-1) | 0 (0-2) | (0-27) | (0-18) | (0-23) | 1,112 |
| 8^th^ year | 2.0 | 0.7 | 1.3 | 1 (0-2) | 1 (0-1) | 0 (0-1) | (0-69) | (0-68) | (0-32) | 1,004 |
| 9^th^ year | 1.9 | 0.7 | 1.2 | 1 (0-2) | 1 (0-1) | 0 (0-1) | (0-40) | (0-12) | (0-40) | 904 |
| 10^th^ year | 1.8 | 0.7 | 1.2 | 1 (0-2) | 0 (0-1) | 0 (0-1) | (0-36) | (0-17) | (0-36) | 795 |
| 11^th^ year | 1.8 | 0.7 | 1.1 | 1 (0-2) | 1 (0-1) | 0 (0-1) | (0-91) | (0-19) | (0-91) | 679 |
| 12^th^ year | 1.8 | 0.7 | 1.1 | 1 (0-2) | 0 (0-1) | 0 (0-1) | (0-34) | (0-15) | (0-34) | 591 |
| 13^th^ year | 2.1 | 0.7 | 1.3 | 1 (0-3) | 0 (0-1) | 0 (0-1) | (0-39) | (0-11) | (0-38) | 508 |
| 14^th^ year | 2.4 | 0.8 | 1.5 | 1 (0-2) | 1 (0-1) | 0 (0-1) | (0-134) | (0-11) | (0-123) | 434 |
| 15^th^ year | 1.9 | 0.7 | 1.2 | 1 (0-2) | 1 (0-1) | 0 (0-1) | (0-23) | (0-6) | (0-23) | 347 |
| 16^th^ year | 1.9 | 0.8 | 1.1 | 1 (0-2) | 0 (0-1) | 0 (0-1) | (0-28) | (0-24) | (0-28) | 254 |
| 17^th^ year | 1.8 | 0.8 | 1.0 | 1 (0-2) | 1 (0-1) | 0 (0-1) | (0-32) | (0-10) | (0-30) | 157 |
| We required patients to be alive and have both Inpatient and Outpatient and Accidents and Emergency room data coverage for at least part of the year reported (row in table). Length of stay in hospital during ASO spell was reported in Table 2. Hospital Episode Statistics (HES) data from ASO discharge was available for 1,619 patients, with median (IQR) follow-up of 8.8 (4.8,13.4) years and minimum (maximum) follow-up of 0.0 (17.9) years, where follow-up of 0.0 years was only affecting patients who died at ASO spell who were not included in the table. | | | | | | | | | | |

| **Supplemental Table 11.** Number of days spent at hospital (as inpatient) by years from arterial switch operation (ASO) spell discharge. | | | | | | | | | | |
| --- | --- | --- | --- | --- | --- | --- | --- | --- | --- | --- |
| **Years from ASO spell** | **Total time in hospital (days) as inpatient** | | | | | | | | | **Patient number** |
|  | *mean* | | | *median (IQR)* | | | *range* | | |  |
|  | **overall** | **cardiac** | **other** | **overall** | **cardiac** | **other** | **overall** | **cardiac** | **other** |  |
| 1^st^ year | 3.2 | 1.5 | 1.7 | 1 (0-2) | 0 (0-0) | 0 (0-1) | (0-296) | (0-296) | (0-170) | 1,590 |
| 2^nd^ year | 0.9 | 0.4 | 0.5 | 0 (0-0) | 0 (0-0) | 0 (0-0) | (0-365) | (0-365) | (0-74) | 1,576 |
| 3^rd^ year | 0.4 | 0.1 | 0.3 | 0 (0-0) | 0 (0-0) | 0 (0-0) | (0-45) | (0-43) | (0-30) | 1,478 |
| 4^th^ year | 0.3 | 0.1 | 0.3 | 0 (0-0) | 0 (0-0) | 0 (0-0) | (0-21) | (0-19) | (0-14) | 1,388 |
| 5^th^ year | 0.5 | 0.3 | 0.2 | 0 (0-0) | 0 (0-0) | 0 (0-0) | (0-193) | (0-192) | (0-22) | 1,311 |
| 6^th^ year | 0.3 | 0.1 | 0.2 | 0 (0-0) | 0 (0-0) | 0 (0-0) | (0-27) | (0-20) | (0-26) | 1,198 |
| 7^th^ year | 0.3 | 0.1 | 0.2 | 0 (0-0) | 0 (0-0) | 0 (0-0) | (0-20) | (0-18) | (0-15) | 1,112 |
| 8^th^ year | 0.3 | 0.1 | 0.1 | 0 (0-0) | 0 (0-0) | 0 (0-0) | (0-68) | (0-67) | (0-12) | 1,004 |
| 9^th^ year | 0.2 | 0.1 | 0.2 | 0 (0-0) | 0 (0-0) | 0 (0-0) | (0-34) | (0-7) | (0-34) | 904 |
| 10^th^ year | 0.2 | 0.1 | 0.1 | 0 (0-0) | 0 (0-0) | 0 (0-0) | (0-13) | (0-13) | (0-6) | 795 |
| 11^th^ year | 0.2 | 0.1 | 0.1 | 0 (0-0) | 0 (0-0) | 0 (0-0) | (0-12) | (0-12) | (0-4) | 679 |
| 12^th^ year | 0.2 | 0.1 | 0.1 | 0 (0-0) | 0 (0-0) | 0 (0-0) | (0-15) | (0-9) | (0-15) | 591 |
| 13^th^ year | 0.2 | 0.1 | 0.1 | 0 (0-0) | 0 (0-0) | 0 (0-0) | (0-12) | (0-10) | (0-9) | 508 |
| 14^th^ year | 0.4 | 0.1 | 0.3 | 0 (0-0) | 0 (0-0) | 0 (0-0) | (0-123) | (0-10) | (0-113) | 434 |
| 15^th^ year | 0.2 | 0.1 | 0.1 | 0 (0-0) | 0 (0-0) | 0 (0-0) | (0-6) | (0-2) | (0-5) | 347 |
| 16^th^ year | 0.3 | 0.1 | 0.2 | 0 (0-0) | 0 (0-0) | 0 (0-0) | (0-26) | (0-22) | (0-26) | 254 |
| 17^th^ year | 0.3 | 0.1 | 0.1 | 0 (0-0) | 0 (0-0) | 0 (0-0) | (0-16) | (0-7) | (0-15) | 157 |
| We required patients to be alive and have Inpatient data coverage for at least part of the year reported (row in table). Length of stay in hospital during ASO spell was reported in Table 2. Hospital Episode Statistics (HES) data from ASO discharge was available for 1,619 patients, with median (IQR) follow-up of 8.8 (4.8-13.4) years and minimum (maximum) follow-up of 0.0 (17.9) years, where follow-up of 0.0 years was only affecting patients who died at ASO spell who were not included in the table. | | | | | | | | | | |

| **Supplemental Table 12.** Number of days spent at hospital as outpatient only (no overlap with inpatient spells) by years from arterial switch operation (ASO) spell discharge. | | | | | | | | | | |
| --- | --- | --- | --- | --- | --- | --- | --- | --- | --- | --- |
| **Years from ASO spell** | **Total time in hospital (days) as outpatient (not inpatient)** | | | | | | | | | **Patient number** |
|  | *mean* | | | *median (IQR)* | | | *range* | | |  |
|  | **overall** | **cardiac** | **other** | **overall** | **cardiac** | **other** | **overall** | **cardiac** | **other** |  |
| 1^st^ year | 5.3 | 2.8 | 2.5 | 4 (3-7) | 3 (2-4) | 1 (0-3) | (0-43) | (0-21) | (0-38) | 1,432 |
| 2^nd^ year | 2.3 | 1.0 | 1.3 | 2 (1-3) | 1 (0-1) | 0 (0-2) | (0-52) | (0-8) | (0-45) | 1,498 |
| 3^rd^ year | 1.9 | 0.8 | 1.2 | 1 (1-2) | 1 (0-1) | 0 (0-1) | (0-65) | (0-14) | (0-51) | 1,478 |
| 4^th^ year | 1.9 | 0.7 | 1.2 | 1 (1-2) | 1 (0-1) | 0 (0-1) | (0-69) | (0-8) | (0-63) | 1,388 |
| 5^th^ year | 1.8 | 0.7 | 1.1 | 1 (1-2) | 1 (0-1) | 0 (0-1) | (0-31) | (0-10) | (0-30) | 1,311 |
| 6^th^ year | 1.9 | 0.6 | 1.2 | 1 (0-2) | 1 (0-1) | 0 (0-2) | (0-39) | (0-12) | (0-27) | 1,198 |
| 7^th^ year | 1.7 | 0.6 | 1.1 | 1 (0-2) | 1 (0-1) | 0 (0-1) | (0-24) | (0-8) | (0-23) | 1,112 |
| 8^th^ year | 1.5 | 0.6 | 1.0 | 1 (0-2) | 1 (0-1) | 0 (0-1) | (0-31) | (0-6) | (0-30) | 1,004 |
| 9^th^ year | 1.5 | 0.6 | 0.9 | 1 (0-2) | 1 (0-1) | 0 (0-1) | (0-37) | (0-6) | (0-36) | 904 |
| 10^th^ year | 1.4 | 0.6 | 0.8 | 1 (0-2) | 0 (0-1) | 0 (0-1) | (0-36) | (0-6) | (0-36) | 795 |
| 11^th^ year | 1.4 | 0.6 | 0.8 | 1 (0-2) | 0 (0-1) | 0 (0-1) | (0-91) | (0-7) | (0-91) | 679 |
| 12^th^ year | 1.4 | 0.6 | 0.8 | 1 (0-2) | 0 (0-1) | 0 (0-1) | (0-34) | (0-7) | (0-34) | 591 |
| 13^th^ year | 1.6 | 0.6 | 1.0 | 1 (0-2) | 0 (0-1) | 0 (0-1) | (0-39) | (0-9) | (0-38) | 508 |
| 14^th^ year | 1.7 | 0.7 | 1.0 | 1 (0-2) | 0 (0-1) | 0 (0-1) | (0-42) | (0-7) | (0-42) | 434 |
| 15^th^ year | 1.5 | 0.7 | 0.8 | 1 (0-2) | 1 (0-1) | 0 (0-1) | (0-22) | (0-5) | (0-22) | 347 |
| 16^th^ year | 1.4 | 0.6 | 0.8 | 1 (0-2) | 0 (0-1) | 0 (0-1) | (0-18) | (0-5) | (0-16) | 254 |
| 17^th^ year | 1.4 | 0.7 | 0.7 | 1 (0-2) | 1 (0-1) | 0 (0-1) | (0-16) | (0-4) | (0-15) | 157 |
| We required patients to be alive and have both Inpatient and Outpatient and Accidents and Emergency room data coverage for at least part of the year reported (row in table). Length of stay in hospital during ASO spell was reported in Table 2. Hospital Episode Statistics (HES) data from ASO discharge was available for 1,619 patients, with median (IQR) follow-up of 8.8 (4.8,13.4) years and minimum (maximum) follow-up of 0.0 (17.9) years, where follow-up of 0.0 years was only affecting patients who died at ASO spell who were not included in the table. | | | | | | | | | | |

| **Supplemental Table 13.** Number of days spent at Accidents and Emergency room (A&E) only, by years from arterial switch operation (ASO) spell discharge. | | | | | | | | | | |
| --- | --- | --- | --- | --- | --- | --- | --- | --- | --- | --- |
| **Years from ASO spell** | **Total time in hospital (days) A&E only** | | | | | | | | | **Patient number** |
|  | *mean* | | | *median (IQR)* | | | *range* | | |  |
|  | **overall** | **cardiac** | **other** | **overall** | **cardiac** | **other** | **overall** | **cardiac** | **other** |  |
| 1^st^ year | 0.6 | 0.0 | 0.6 | 0 (0-1) | 0 (0-0) | 0 (0-1) | (0-11) | (0-2) | (0-11) | 1,078 |
| 2^nd^ year | 0.5 | 0.0 | 0.5 | 0 (0-1) | 0 (0-0) | 0 (0-1) | (0-7) | (0-1) | (0-7) | 1,142 |
| 3^rd^ year | 0.4 | 0.0 | 0.4 | 0 (0-1) | 0 (0-0) | 0 (0-1) | (0-7) | (0-1) | (0-7) | 1,131 |
| 4^th^ year | 0.3 | 0.0 | 0.3 | 0 (0-1) | 0 (0-0) | 0 (0-1) | (0-7) | (0-1) | (0-7) | 1,134 |
| 5^th^ year | 0.3 | 0.0 | 0.3 | 0 (0-0) | 0 (0-0) | 0 (0-0) | (0-5) | (0-0) | (0-5) | 1,154 |
| 6^th^ year | 0.2 | 0.0 | 0.2 | 0 (0-0) | 0 (0-0) | 0 (0-0) | (0-5) | (0-1) | (0-5) | 1,120 |
| 7^th^ year | 0.2 | 0.0 | 0.2 | 0 (0-0) | 0 (0-0) | 0 (0-0) | (0-4) | (0-2) | (0-4) | 1,112 |
| 8^th^ year | 0.2 | 0.0 | 0.2 | 0 (0-0) | 0 (0-0) | 0 (0-0) | (0-4) | (0-3) | (0-4) | 1,004 |
| 9^th^ year | 0.2 | 0.0 | 0.2 | 0 (0-0) | 0 (0-0) | 0 (0-0) | (0-5) | (0-1) | (0-5) | 904 |
| 10^th^ year | 0.2 | 0.0 | 0.2 | 0 (0-0) | 0 (0-0) | 0 (0-0) | (0-6) | (0-1) | (0-6) | 795 |
| 11^th^ year | 0.2 | 0.0 | 0.2 | 0 (0-0) | 0 (0-0) | 0 (0-0) | (0-3) | (0-1) | (0-3) | 679 |
| 12^th^ year | 0.2 | 0.0 | 0.2 | 0 (0-0) | 0 (0-0) | 0 (0-0) | (0-5) | (0-1) | (0-5) | 591 |
| 13^th^ year | 0.2 | 0.0 | 0.2 | 0 (0-0) | 0 (0-0) | 0 (0-0) | (0-3) | (0-1) | (0-3) | 508 |
| 14^th^ year | 0.2 | 0.0 | 0.2 | 0 (0-0) | 0 (0-0) | 0 (0-0) | (0-4) | (0-0) | (0-4) | 434 |
| 15^th^ year | 0.2 | 0.0 | 0.2 | 0 (0-0) | 0 (0-0) | 0 (0-0) | (0-5) | (0-1) | (0-5) | 347 |
| 16^th^ year | 0.2 | 0.0 | 0.2 | 0 (0-0) | 0 (0-0) | 0 (0-0) | (0-3) | (0-0) | (0-3) | 254 |
| 17^th^ year | 0.2 | 0.0 | 0.2 | 0 (0-0) | 0 (0-0) | 0 (0-0) | (0-6) | (0-1) | (0-5) | 157 |
| We required patients to be alive and have both Inpatient and Outpatient and A&E data coverage for at least part of the year reported (row in table). Length of stay in hospital during ASO spell was reported in Table 2. Hospital Episode Statistics (HES) data from ASO discharge was available for 1,619 patients, with median (IQR) follow-up of 8.8 (4.8-13.4) years and minimum (maximum) follow-up of 0.0 (17.9) years, where follow-up of 0.0 years was only affecting patients who died at ASO spell who were not included in the table. | | | | | | | | | | |

| **Supplemental Table 14.** Number of days spent at hospital (as either inpatient, outpatient or Accidents and Emergency room) by years from arterial switch operation (ASO) spell discharge for the 183 patients with at least one known cardiac reintervention. | | | | | | | | | | |
| --- | --- | --- | --- | --- | --- | --- | --- | --- | --- | --- |
| **Years from ASO spell** | **Total time in hospital (days)** | | | | | | | | | **Patient number** |
|  | *mean* | | | *median (IQR)* | | | *range* | | |  |
|  | **overall** | **cardiac** | **other** | **overall** | **cardiac** | **other** | **overall** | **cardiac** | **other** |  |
| 1^st^ year | 23.0 | 16.1 | 6.9 | 14 (7-25) | 7 (4-15) | 4 (1-9) | (0-264) | (0-259) | (0-79) | 96 |
| 2^nd^ year | 6.4 | 2.8 | 3.5 | 4 (2-7) | 2 (1-3) | 2 (0-4) | (0-56) | (0-23) | (0-33) | 100 |
| 3^rd^ year | 4.0 | 1.6 | 2.4 | 3 (2-5) | 1 (1-2) | 1 (0-3) | (0-17) | (0-14) | (0-15) | 110 |
| 4^th^ year | 3.8 | 1.6 | 2.2 | 2 (1-4) | 1 (1-2) | 1 (0-2) | (0-44) | (0-27) | (0-17) | 116 |
| 5^th^ year | 6.0 | 3.7 | 2.3 | 2 (1-5) | 1 (1-2) | 1 (0-3) | (0-197) | (0-194) | (0-24) | 122 |
| 6^th^ year | 3.9 | 1.5 | 2.4 | 2 (1-5) | 1 (0-1) | 1 (0-3) | (0-66) | (0-32) | (0-34) | 120 |
| 7^th^ year | 3.3 | 1.2 | 2.1 | 2 (1-4) | 1 (0-1) | 1 (0-3) | (0-27) | (0-18) | (0-21) | 121 |
| 8^th^ year | 3.8 | 1.8 | 1.9 | 2 (1-4) | 1 (0-1) | 1 (0-3) | (0-69) | (0-68) | (0-32) | 111 |
| 9^th^ year | 3.4 | 1.3 | 2.1 | 2 (1-4) | 1 (0-2) | 0 (0-2) | (0-39) | (0-12) | (0-38) | 104 |
| 10^th^ year | 3.2 | 1.5 | 1.7 | 2 (1-3) | 1 (0-2) | 0 (0-2) | (0-36) | (0-14) | (0-36) | 92 |
| 11^th^ year | 3.8 | 1.4 | 2.3 | 1 (1-3) | 1 (0-1) | 0 (0-1) | (0-91) | (0-19) | (0-91) | 86 |
| 12^th^ year | 3.6 | 1.6 | 2.0 | 2 (1-4) | 1 (0-1) | 0 (0-2) | (0-27) | (0-14) | (0-27) | 79 |
| 13^th^ year | 3.2 | 1.6 | 1.7 | 2 (1-4) | 1 (0-2) | 0 (0-2) | (0-28) | (0-11) | (0-28) | 70 |
| 14^th^ year | 3.2 | 1.2 | 2.0 | 2 (1-4) | 1 (0-2) | 0 (0-2) | (0-42) | (0-9) | (0-42) | 64 |
| 15^th^ year | 2.0 | 1.0 | 1.0 | 1 (1-3) | 1 (0-1) | 0 (0-1) | (0-12) | (0-4) | (0-12) | 49 |
| 16^th^ year | 1.5 | 1.1 | 0.3 | 1 (0-3) | 1 (0-2) | 0 (0-0) | (0-6) | (0-6) | (0-3) | 36 |
| 17^th^ year | 2.1 | 1.5 | 0.6 | 2 (0-2) | 1 (0-2) | 0 (0-1) | (0-11) | (0-10) | (0-5) | 22 |
| We required patients to be alive and have both Inpatient and Outpatient and Accidents and Emergency room data coverage for at least part of the year reported (row in table). | | | | | | | | | | |

## **Matched samples description**

| **Supplemental Table 15.** Age distribution over pooling and matched samples of BAS+ASO to primary ASO patients. | | | | | | | | | | | | |
| --- | --- | --- | --- | --- | --- | --- | --- | --- | --- | --- | --- | --- |
|  | **N** | **Min** | **p1** | **p5** | **p10** | **p25** | **p50** | **p75** | **p90** | **p95** | **p99** | **Max** |
| **Pooling sample** |  |  |  |  |  |  |  |  |  |  |  |  |
| Primary ASO | 690 | 0.5 | 1.5 | 2.5 | 4.4 | 6.5 | 8.5 | 11.5 | 16.5 | 18.5 | 20.5 | 20.5 |
| BAS+ASO | 939 | 0.0 | 0.3 | 0.5 | 0.5 | 0.5 | 1.5 | 2.1 | 4.5 | 10.5 | 16.8 | 20.9 |
| **Matched sample** | | | | | | | | | | | | |
| Primary ASO | 174 | 0.5 | 1.4 | 2.4 | 2.5 | 3.5 | 5.5 | 10.5 | 15.5 | 17.5 | 20.5 | 20.5 |
| BAS+ASO | 174 | 0.5 | 1.5 | 1.9 | 2.5 | 2.5 | 5.5 | 10.5 | 15.5 | 17.5 | 20.5 | 20.9 |
| The pooling sample consists of patients with first BAS/ASO before 3 weeks of life and valid age-sex-weight information.  ASO, arterial switch operation; BAS, balloon atrial septostomy. | | | | | | | | | | | | |

| **Supplemental Table 16.** Age-sex Z-score distribution over pooling and matched samples of BAS+ASO to primary ASO patients. | | | | | | | | | | | | |
| --- | --- | --- | --- | --- | --- | --- | --- | --- | --- | --- | --- | --- |
|  | **N** | **Min** | **p1** | **p5** | **p10** | **p25** | **p50** | **p75** | **p90** | **p95** | **p99** | **Max** |
| **Pooling sample** |  |  |  |  |  |  |  |  |  |  |  |  |
| Primary ASO | 690 | -5.5 | -3.0 | -2.3 | -1.8 | -1.0 | -0.3 | 0.4 | 1.0 | 1.6 | 2.4 | 9.6 |
| BAS+ASO | 939 | -5.3 | -3.5 | -2.3 | -1.7 | -1.0 | -0.3 | 0.4 | 1.0 | 1.4 | 2.2 | 3.3 |
| **Matched sample** | |  |  |  |  |  |  |  |  |  |  |  |
| Primary ASO | 174 | -2.8 | -2.6 | -1.9 | -1.6 | -1.0 | -0.4 | 0.3 | 1.0 | 1.6 | 2.2 | 2.6 |
| BAS+ASO | 174 | -3.5 | -3.2 | -2.6 | -2.0 | -1.1 | -0.3 | 0.4 | 0.9 | 1.2 | 2.1 | 2.2 |
| The pooling sample consists of patients with first BAS/ASO before 3 weeks of life and valid age-sex-weight information.  ASO, arterial switch operation; BAS, balloon atrial septostomy. | | | | | | | | | | | | |

| **Supplemental Table 17.** Yearly distribution over pooling and matched samples of BAS+ASO to primary ASO patients. | | | | | | | | | | | | |
| --- | --- | --- | --- | --- | --- | --- | --- | --- | --- | --- | --- | --- |
|  | **N** | **Min** | **p1** | **p5** | **p10** | **p25** | **p50** | **p75** | **p90** | **p95** | **p99** | **Max** |
| **Pooling sample** |  |  |  |  |  |  |  |  |  |  |  |  |
| Primary ASO | 690 | 2000 | 2000 | 2000 | 2001 | 2003 | 2008 | 2012 | 2015 | 2016 | 2016 | 2016 |
| BAS+ASO | 939 | 2000 | 2000 | 2001 | 2002 | 2004 | 2008 | 2012 | 2015 | 2016 | 2016 | 2016 |
| **Matched sample** | |  |  |  |  |  |  |  |  |  |  |  |
| Primary ASO | 174 | 2000 | 2000 | 2000 | 2002 | 2003 | 2008 | 2011 | 2014 | 2015 | 2016 | 2016 |
| BAS+ASO | 174 | 2000 | 2000 | 2000 | 2001 | 2004 | 2008 | 2011 | 2014 | 2015 | 2016 | 2016 |
| The pooling sample consists of patients with first BAS/ASO before 3 weeks of life and valid age-sex-weight information.  ASO, arterial switch operation; BAS, balloon atrial septostomy. | | | | | | | | | | | | |

**SUPPLEMENTAL REFERENCES**

1. NICOR CCAD. Available at: https://nicor4.nicor.org.uk/chd/an_paeds.nsf/vwcontent/home.

2. Rogers L, Brown KL, Franklin RC, et al. Improving Risk Adjustment for Mortality After Pediatric Cardiac Surgery: The UK PRAiS2 Model. Annals of Thoracic Surgery 2017;104:211–219.

3. Pujol FE, Pagel C, Brown KL, et al. Linkage of National Congenital Heart Disease Audit data to hospital, critical care and mortality national data sets to enable research focused on quality improvement. BMJ Open 2022;12:e057343.

4. Pujol FE, Franklin RC, Crowe S, et al. Transfer of congenital heart patients from paediatric to adult services in England. Heart 2022:heartjnl-2022-321085. Available at:

5. Sarris GE, Balmer C, Bonou P, et al. Clinical guidelines for the management of patients with transposition of the great arteries with intact ventricular septum. Cardiol Young 2017;27:530–569.
